# Rational Design of a Carrier Protein for the Production of Recombinant Toxic Peptides in *Escherichia coli*

**DOI:** 10.1371/journal.pone.0146552

**Published:** 2016-01-25

**Authors:** Katia Pane, Lorenzo Durante, Elio Pizzo, Mario Varcamonti, Anna Zanfardino, Valeria Sgambati, Antimo Di Maro, Andrea Carpentieri, Viviana Izzo, Alberto Di Donato, Valeria Cafaro, Eugenio Notomista

**Affiliations:** 1 Department of Biology, Università degli Studi di Napoli Federico II, Napoli, Italy; 2 Department of Environmental, Biological and Pharmaceutical Sciences and Technologies, Seconda Università di Napoli, Caserta, Italy; 3 Department of Chemical Sciences, Università degli Studi di Napoli Federico II, Napoli, Italy; 4 Department of Medicine and Surgery, Università degli Studi di Salerno, Baronissi, Italy; Centro Nacional de Biotecnologia - CSIC / CIF Q2818002D, SPAIN

## Abstract

Commercial uses of bioactive peptides require low cost, effective methods for their production. We developed a new carrier protein for high yield production of recombinant peptides in *Escherichia coli* very well suited for the production of toxic peptides like antimicrobial peptides. GKY20, a short antimicrobial peptide derived from the C-terminus of human thrombin, was fused to the C-terminus of Onconase, a small ribonuclease (104 amino acids), which efficiently drove the peptide into inclusion bodies with very high expression levels (about 200–250 mg/L). After purification of the fusion protein by immobilized metal ion affinity chromatography, peptide was obtained by chemical cleavage in diluted acetic acid of an acid labile Asp-Pro sequence with more than 95% efficiency. To improve peptide purification, Onconase was mutated to eliminate all acid labile sequences thus reducing the release of unwanted peptides during the acid cleavage. Mutations were chosen to preserve the differential solubility of Onconase as function of pH, which allows its selective precipitation at neutral pH after the cleavage. The improved carrier allowed the production of 15–18 mg of recombinant peptide per liter of culture with 96–98% purity without the need of further chromatographic steps after the acid cleavage. The antimicrobial activity of the recombinant peptide, with an additional proline at the N-terminus, was tested on Gram-negative and Gram-positive strains and was found to be identical to that measured for synthetic GKY20. This finding suggests that N-terminal proline residue does not change the antimicrobial properties of recombinant (P)GKY20. The improved carrier, which does not contain cysteine and methionine residues, Asp-Pro and Asn-Gly sequences, is well suited for the production of peptides using any of the most popular chemical cleavage methods.

## Introduction

Peptides of length between five and fifty residues have many applications in research, diagnostic, medicine (e.g. antimicrobial [1, 2], anti-inflammatory [3], anti-cancer [4] and cell penetrating peptides [5] etc.) and industry (e.g. metal surface-binding [6] and graphite-binding peptides [7]). Solid phase chemical synthesis is well suited to obtain small amounts of peptides but it could be rather expensive when peptides longer than 30 amino acids or high quantities of peptide are required. On the other hand, recombinant production of peptides [8–11] entails several difficulties. For example, expression of some peptides can be toxic for the host and several peptides are very sensitive to proteases thus leading to low yields. These difficulties can be circumvented by fusing the desired peptide to a carrier protein that can protect peptides from proteases, neutralize possible toxic effects and provide a convenient route for their purification. However, also fusion protein strategy has two main drawbacks. Firstly, the fusion protein has to be cleaved and peptide needs to be separated from the carrier, secondly, the desired peptide usually represents just a small percentage of the purified fusion protein. As for the cleavage of the peptide, it can be released from the fusion protein by enzymatic or chemical cleavage at a site suitably introduced at the carrier-peptide junction ([10] and references therein). The efficiency and specificity of the cleavage is the main bottleneck of fused peptide production. Usually enzymatic proteolysis by factor Xa [12, 13], enterokinase [14] and thrombin [15, 16], the most used enzymes, is less efficient than chemical cleavage [17–19]. The lower efficiency of enzymatic methods is generally attributed to unfavorable surrounding residues and/or to steric hindrance which make the cleavage sites not fully accessible. Furthermore, enzymes usually require non-denaturing reaction conditions to work properly. Thus, enzymatic proteolysis cannot be used when the fusion protein is partly or completely insoluble. On the other hand, the most popular cleavage reagents, like cyanogen bromide (CNBr), formic acid and hydroxylamine, which cleave Met-X, Asp-Pro and Asn-Gly peptide bonds [10, 20, 21], respectively, often produce unwanted cleavages and side-chain modifications due to the harsh reaction conditions needed for the cleavage [22–28]. For example, cleavage of Asp-Pro sequences, usually performed at 60°C in 50–70% formic acid, often results in unspecific hydrolysis, formylation and oxidation reactions of side chains [28].

Here, we describe the rational development of a new carrier protein for high yield production of recombinant peptides in *Escherichia coli* very well suited for toxic peptides like antimicrobial peptides [10]. The denatured form of Onconase (ONC), a RNase from *Rana pipiens* [29], was chosen as starting point to design the carrier protein. ONC is a very well suited partner for several reasons: (i) it can be expressed at very high levels in inclusion bodies (about 200–250 mg/L in terrific broth); (ii) usually no soluble ONC can be detected in growth cultures thus minimizing the risk of toxic effects of the ONC-peptide fusion proteins; (iii) it is a very small protein (104 aa) thus allowing high yields of the peptides after the cleavage; (iv) the solubility of denatured Onconase is pH dependent—the denatured protein is soluble only at pH <4 –thus allowing the purification of peptides soluble at pH 7 by selective precipitation of the carrier. Moreover, ONC does not contain Asp-Pro and Asn-Gly sequences and the mutant (M23L)-ONC [30] does not contain internal methionine residues, therefore it is compatible with all common chemical cleavage strategies.

Starting from mutant (M23L)-ONC we have developed an improved carrier protein bearing 15 mutations and an improved cleavage procedure of Asp-Pro peptide bonds that does not require formic acid.

We tested the efficiency of our strategy by producing GKY20, a short cationic antimicrobial peptide derived from the C-terminus of human thrombin [31]. Like other similar antimicrobial peptides, GKY20 is endowed with broad spectrum antimicrobial activity being active both on Gram-positive and Gram-negative bacteria. Its bactericidal activity has been attributed to its capacity to induce membrane lysis. Moreover, it shows potent anti-inflammatory activity due to its ability to bind LPS thus inhibiting macrophage responses to these components of the outer membrane [32]. Noteworthy, GKY20 is active on different strains of *E*. *coli* with a minimal inhibitory concentration (MIC) lower than 10 μM [33]. In spite of this high toxicity we were able to prepare recombinant GKY20 in *E*. *coli* with final yield of about 15–20 mg per L of culture and purity higher than 96–98%.

## Results and Discussion

### Expression vector design: ONC-(P)GKY20 fusion protein

**Fig 1:**
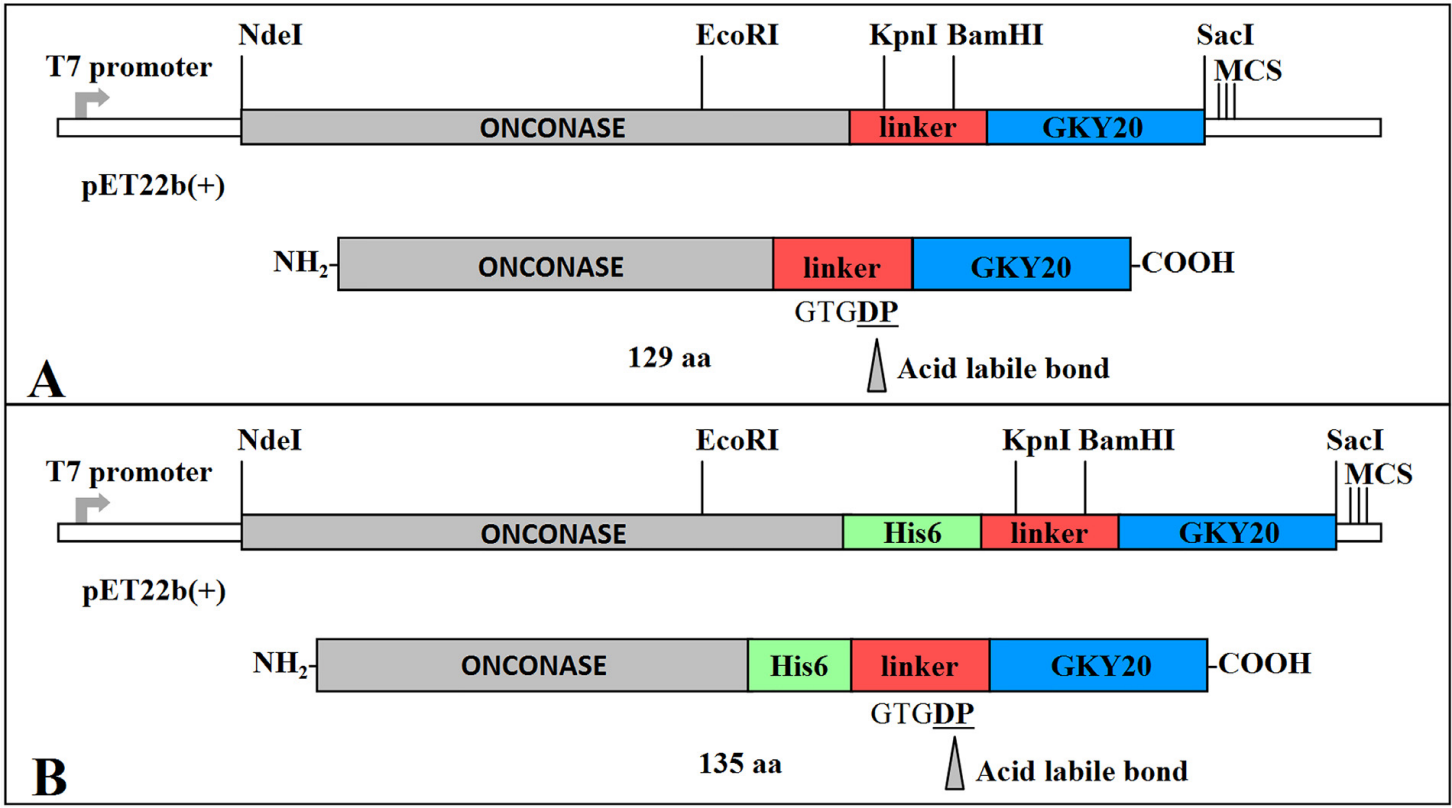
Schematic representation of expression vectors and recombinant proteins. Fusion proteins without (A) and with (B) His_6_-Tag. The main restriction enzyme sites, *NdeI*, *EcoRI*, *KpnI*, *BamHI* and *SacI* were reported. MCS: multicloning site. Onconase: carrier protein (grey). Linker: DNA sequence coding for GTGDP amino acid residues (red). GKY20: human Thrombin derived peptide (blue).

Native ONC is a small protein (104 aa) stabilized by four disulfide bridges. When expressed in *E*. *coli*, wild type ONC and its mutants are produced at high yield as inclusion bodies [29, 30]. We have previously shown that once extracted by denaturation using guanidine chloride and dialyzed in acetic acid 0.1 M the denatured form is very soluble, whereas at pH 7 it is prone to aggregation and efficient renaturation can only be achieved by a special procedure based on reversible blocking of cysteine residues [29]. In order to use denatured ONC as carrier for the production of recombinant peptide GKY20, we prepared a chimeric open reading frame including codons 1–103 of (M23L)-ONC [34], five codons coding for a linker sequence (GTGDP) containing the formic acid cleavable aspartyl-prolyl dipeptide, and a sequence coding for GKY20 (Fig 1A and S1 File, panel A). This sequence was obtained by changing six codons in the human coding sequence to better match *E*. *coli* codon usage (S1 File, panel C). It should be reminded that ONC does not contain Asp-Pro and Asn-Gly sequences (S1 File, panel A). Moreover, mutant (M23L)-ONC does not contain any internal methionine residue. Thus, mutant (M23L)-ONC represents an ideal scaffold for the chimeric construct because it is not cleaved by the usual chemical cleavage strategies.

The linker sequence was chosen both to introduce a flexible region into the chimeric protein between the ONC moiety and GKY20 and to introduce two restriction enzyme sites, *KpnI* (codons coding for amino acids GT) and *BamHI* (codons coding for amino acids GDP). Moreover, C-terminal cysteine of the ONC moiety (Cys104) was deleted to avoid the presence of an oxidation prone residue near to the cleavage site. The synthetic construct and the corresponding recombinant protein were named ONC-(P)GKY20 to underline that, by acid cleavage, the recombinant protein releases peptide (P)GKY20 i.e. peptide GKY20 with an additional proline at the N-terminus. The construct was cloned into pET22b(+) plasmid between *NdeI* and *SacI* restriction sites (Fig 1A) and expressed into BL21(DE3) *E*. *coli* cells. Expression of recombinant protein was obtained as described in Materials and Methods.

**Fig 2:**
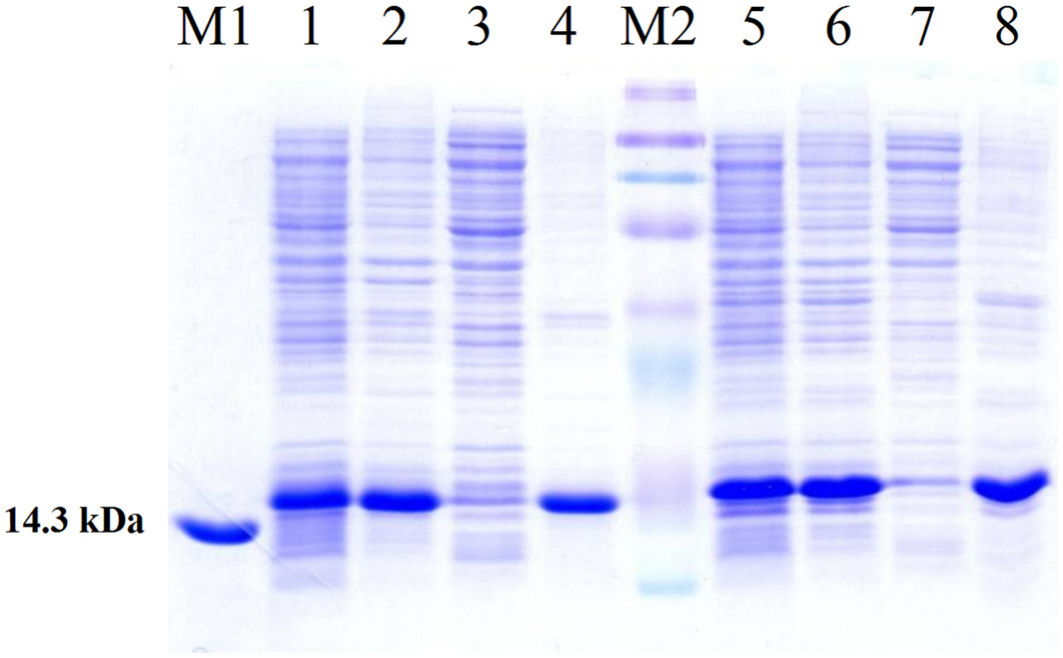
Protein expression. SDS-PAGE analysis of ONC-(P)GKY20 and ONC-DCless-H6-(P)GKY20 fusion proteins. Lane M1: marker (14.3 kDa). Lanes 1–4, ONC-(P)GKY20 protein: lane 1, cell lysate of induced culture; lane 2, insoluble fraction after cell lysis; lane 3, soluble fraction after cell lysis; lane 4, purified ONC-(P)GKY20 recombinant protein after dialysis against 0.1 M acetic acid. Lane M2: marker (8-12-20-30-45-60-100-220 kDa proteins). Lanes 5–8, ONC-DCless-H6-(P)GKY20 fusion protein: lane 5, cell lysate of induced culture; lane 6, insoluble fraction after cell lysis; lane 7, soluble fraction after cell lysis; lane 8, inclusion bodies after triton/urea wash.

SDS-PAGE analysis of induced cultures (Fig 2) showed high expression levels of ONC-(P)GKY20 (200–250 mg/L) as inclusion bodies. The recombinant protein was partially purified by denaturation of the inclusion bodies and dialysis in 0.1 M acetic acid pH 2.9 that causes the precipitation of most of the *E*. *coli* proteins. As shown in Fig 2 (lane 4), after dialysis the soluble fraction contained less than 15–20% of *E*. *coli* proteins as determined by SDS-PAGE densitometry scan. This sample was used to optimize the acidic cleavage of the DP sequence as described in the next section.

### Chemical cleavage optimization of aspartyl-prolyl bonds in ONC-(P)GKY20 fusion protein

The cleavage of Asp-Pro sequences is usually performed at temperatures in the range 55–80°C in 50–75% formic acid. However, these reaction conditions often produce unwanted side reactions like formylation and unspecific hydrolysis [22–28].

**Fig 3:**
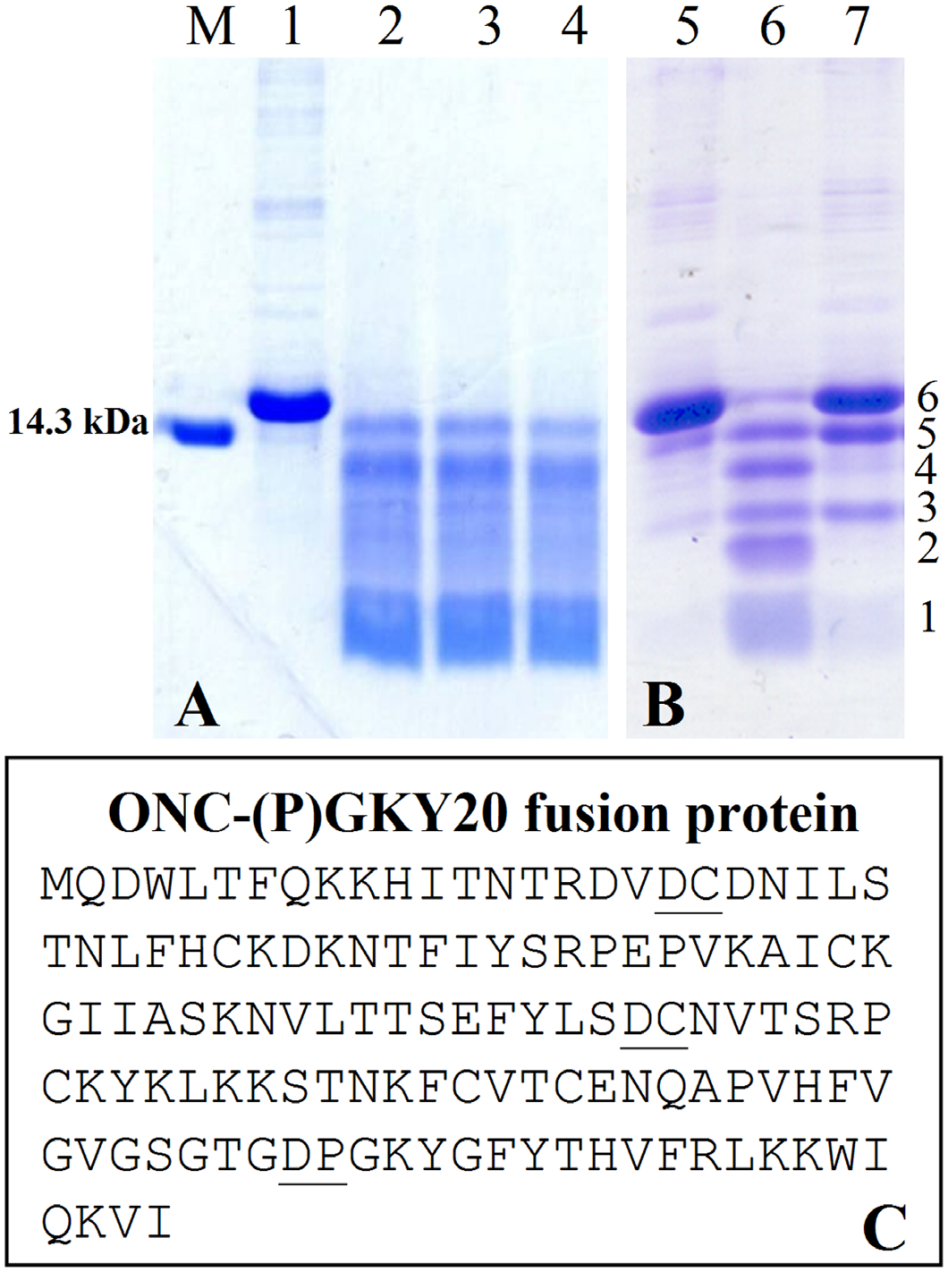
Hydrolysis pattern of ONC-(P)GKY20 protein. Samples were cleaved at acid pH for 24 h at 60°C and analyzed on 20% SDS-PAGE. Lane M: marker (14.3 kDa). Lanes 1, 5: purified fusion protein. (A) ONC-(P)GKY20 protein hydrolyzed in 50% (lane 2), 60% (lane 3) e 70% (lane 4) formic acid (60°C, 24 h). (B) ONC-(P)GKY20 protein hydrolyzed in 0.1 M acetic acid, pH 2.9, (lane 6) and in 0.1 M acetic acid adjusted at pH 2.0 with HCl (lane 7); the N—terminal sequences of the fragments obtained by hydrolysis of ONC-(P)GKY20 fusion protein (lane 6) were as follow: fragment 1, PGK [(P)GKY20 peptide)] and MQD (N-terminal peptide, less represented); fragment 2, X(Cys)NVT (D67-C68 cleavage site); fragment 3, X(Cys)NVT (D67-C68 cleavage site) and MQD (N-terminal peptide, less represented); fragment 4, X(Cys)DNI (D18-C19 cleavage site); fragments 5 and 6, MQD (N-terminal region) which represent the carrier protein and uncleaved fusion protein respectively. (C) Amino acid sequence of ONC-(P)GKY20 fusion protein; the main cleavage sites are underlined.

In order to find less harsh cleavage conditions, thermal incubations of ONC-(P)GKY20 fusion protein were carried out in different acid solutions: 50%, 60% or 70% formic acid (Fig 3A), 0.1 M acetic acid (pH 2.9), 0.1 M acetic acid adjusted at pH 2.0 with HCl (Fig 3B). Samples were incubated at 50° or 60°C for 3, 6, 9, 15 or 24 h. With the exception of 0.1 M acetic acid pH 2.9 (Fig 3B), all the acidic solutions resulted in the efficient hydrolysis (>90%) of the recombinant fusion protein by incubating samples at 60°C for times ≥ 15 h, but with a significant difference: in the case of the acetic acid solution at pH 2.0, a specific cleavage pattern with the formation of six discrete polypeptide bands was visible on SDS-PAGE (Fig 3B), whereas in the case of formic acid, in addition to the bands observed in the case of the acetic acid/HCl hydrolysis, a smear was clearly evident suggesting the occurrence of unspecific cleavages (Fig 3A).

This experiment indicated that it is possible to cleave the recombinant protein without using high concentrations of organic acids, but unfortunately they also revealed two unexpected problems: i) mild acidic hydrolysis cleaved ONC-(P)GKY20 in several fragments (Fig 3B) in spite of the fact that it contains a single Asp-Pro sequence between ONC and GKY20; ii) some *E*. *coli* proteins, present in small amount as contaminants in the inclusion bodies, also underwent cleavage releasing shorter fragments as evidenced by the disappearance of high molecular weight bands.

N-terminal sequencing of the fragments separated by SDS-PAGE (Fig 3B), allowed to identify three main sites of cleavage in the fusion protein. In addition to the expected cleavage at the Asp-Pro sequence in the linker sequence, which releases the desired peptide (P)GKY20, two unexpected sites of hydrolysis were identified: Asp18-Cys19 and Asp67-Cys68. An incomplete hydrolysis at these sites accounts for the six bands observed in the SDS-PAGE (Fig 3B). Band 1 contained (P)GKY20 and a peptide corresponding to the N-terminus of ONC (including initial methionine). Intermediate bands were due to fragments starting at Cys19 and Cys68. Bands 5 and 6 contained fragments starting at the N-terminal sequence of ONC. On the basis of their migration they are likely the uncleaved fusion protein (band 6) and the ONC moiety resulting from a single cleavage at the Asp-Pro sequence (band 5). To the best of our knowledge this is the first report of efficient acid induced cleavage at Asp-Cys sequences.

On the basis of these observations we concluded that the acid cleavage with 0.1 M acetic acid pH 2.0 of ONC-(P)GKY20 fusion protein could not be used to obtain pure (P)GKY20 peptide. Thus, we decided to try to improve the carrier sequence (i) to overcome the undesired cleavages we have observed, and (ii) to avoid the formation of the short fragments derived from the hydrolysis of contaminant *E*. *coli* proteins.

### Improvement of the carrier sequence: first generation mutants

Improvement of the Onconase carrier was performed by a trial and error procedure. For simplicity in Table 1 several different versions of the carrier are divided into "first generation” and “second generation” fusion proteins.

**Table 1:** Expression vectors, fusion proteins and peptides.

Expression vectors	Fusion proteins	Linker and Cleavage site	Peptides	Peptide (N-terminal end)
***First generation fusion proteins***				
*pET22b(+)/ONC-(P)GKY20*	ONC-(P)GKY20^a^	GTG**DP** *GKY*..	(P)GKY20	P-GKY…
*pET22b(+)/ONC(YY)-(P)GKY20*	ONC(YY)-(P)GKY20^b^	GTG**DP** *GKY*..	(P)GKY20	P-GKY…
*pET22b(+)/ONC(EYEY)-(P)GKY20*	ONC(EYEY)-(P)GKY20^c^	GTG**DP** *GKY*..	(P)GKY20	P-GKY…
*pET22b(+)/ONC(EYEY)-H6-(P)GKY20*	ONC(EYEY)-H6-(P)GKY20^d^	GTG**DP** *GKY*..	(P)GKY20	P-GKY…
***Second generation fusion proteins***				
*pET22b(+)/ONC-DCless-H6-(P)GKY20*	ONC-DCless-H6-(P)GKY20^e^	GTG**DP** *GKY*..	(P)GKY20	P-GKY…
*pET22b(+)/ONC-DCless-H6-(PM)GKY20*	ONC-DCless-H6-(PM)GKY20^e^	GTG**DP**M *GKY*..	(PM)GKY20	PM-GKY…
-	-	-	GKY20	GKY…

^a^ ONC: Onconase mutant-M23L,C104 delete;

^b^ ONC(YY): Onconase mutant-M23L,C19Y,C68Y,C104 delete;

^c^ ONC(EYEY): Onconase mutant-M23L,D18E,C19Y,D67E,C68Y,C104 delete;

^d^ ONC(EYEY)-H6: Onconase mutant-M23L,D18E,C19Y,D67E,C68Y,C104 delete; H6: Hexa-histidine tag at the C-terminal side of the onconase carrier.

^e^ ONC-DCless-H6: Onconase mutant-M23L,D2E,D16E,D18E,C19Y,D20E,C30Y,D32E,C48L,D67E,C68Y,C75Y,C87I,C90I,C104 delete; H6: Hexa-histidine tag at the C-terminal side of the onconase carrier.

The mutations introduced into the first generation proteins were chosen mainly to remove the unexpected Asp-Cys cleavage sites and to obtain a high purity fusion protein in order to reduce the release of unwanted peptides during the acid cleavage step from contaminant *E*. *coli* proteins.

As for unexpected Asp-Cys cleavage sites, sequences Asp-Cys were mutated to Asp-Tyr and Glu-Tyr. Mutations were chosen to preserve the propensity of denatured ONC to form inclusion bodies and to aggregate at pH 7. Since protein solubility and propensity to form aggregates, like inclusion bodies, are generally related to net charge, hydrophobicity and to the presence of amphipatic secondary structures [35, 36], the amino acid substitutions were chosen in order to preserve these properties. Therefore, aspartic acid residues were replaced with glutamic acid residues in order to preserve the net charge. Cysteine residues were replaced with tyrosine residues as in several hydrophobicity scales these two residues show very similar hydrophobicity scores [37]. Moreover, the replacement of Asp-Cys with Glu-Tyr allows to keep unchanged secondary structure propensity since the replacement of aspartate with glutamate decreases the preference for loop structures, whereas the replacement of cysteine with tyrosine increases the preference for loop structures. Furthermore, the mutant bearing the two Glu-Tyr sequences was also prepared with a six histidine-tag sequence (H6) inserted between the ONC moiety and the flexible linker GTGDP (Fig 1B).

**Fig 4:**
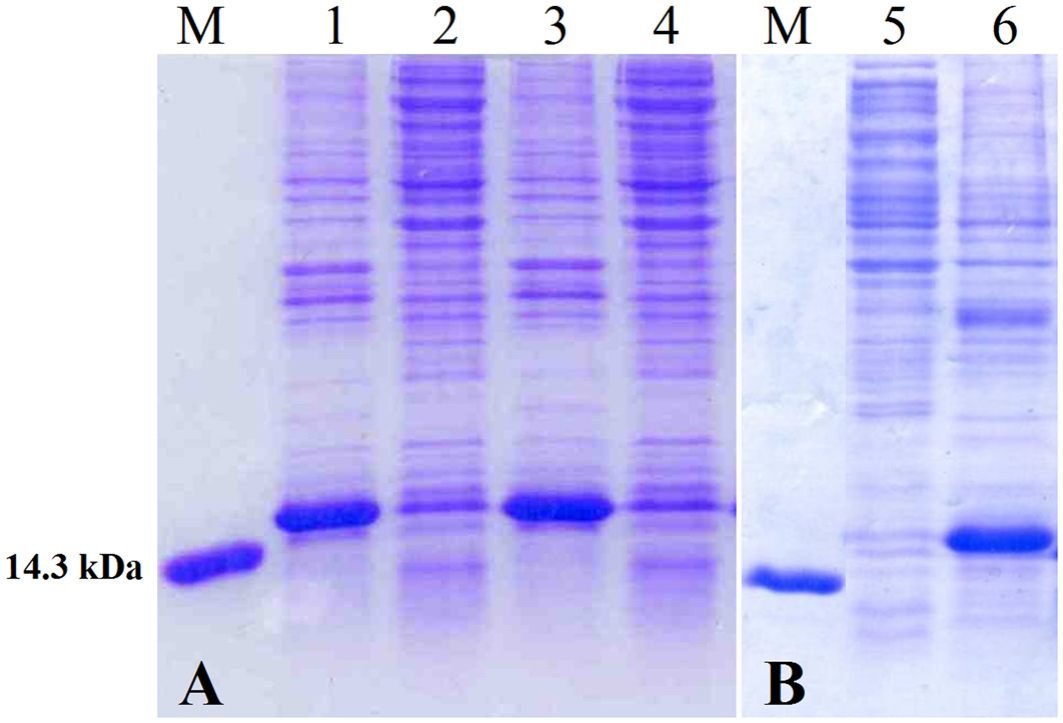
Protein expression of first generation mutants. (A) SDS-PAGE analysis of insoluble (lanes 1, 3) and soluble (lanes 2, 4) fractions after cell lysis of ONC(YY)-(P)GKY20 and ONC(EYEY)-(P)GKY20 fusion proteins, respectively. (B) SDS-PAGE analysis of soluble (lane 5) and insoluble (lane 6) fractions after cell lysis of ONC(EYEY)-H6-(P)GKY20 fusion protein. Lane M: marker (14.3 kDa).

All the recombinant fusion proteins showed high expression levels as inclusion bodies, thus indicating that the chosen mutations did not interfere with the formation of inclusion bodies (Fig 4).

**Fig 5:**
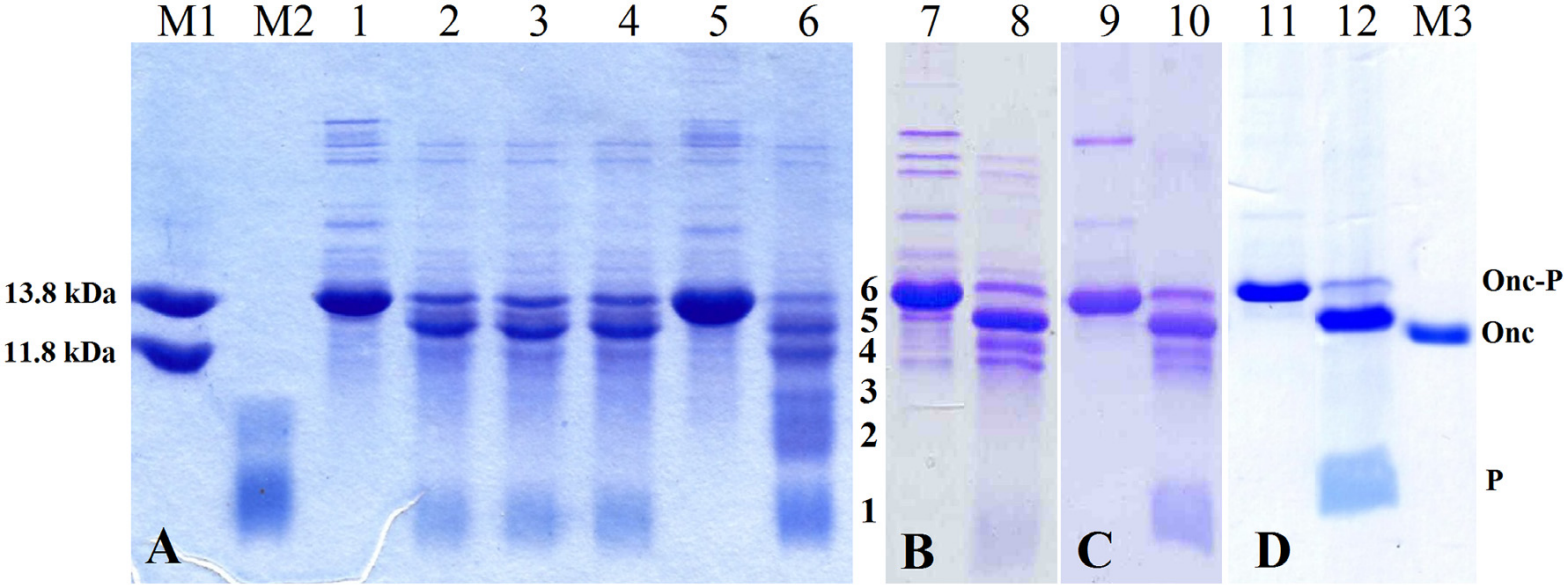
Comparison of hydrolysis patterns of first and second generation mutants. Chemical cleavage was carried out in 0.1 M acetic acid adjusted at pH 2.0 with HCl (60°C, 24 h). Samples were analyzed on 20% SDS-PAGE. Lane M1: markers (13.8 kDa, 11.8 kDa). Lane M2: RK-16 peptide (1961.25 Da). Lane M3: marker (14.3 kDa). (A) ONC(YY)-(P)GKY20 purified fusion protein (lane 1) and hydrolyzed protein (lanes 2–4, three independent experiments). ONC-(P)GKY20 purified protein (lane 5) and cleaved protein (lane 6). (B, C, D) ONC(EYEY)-(P)GKY20, ONC(EYEY)-H6-(P)GKY20 and ONC-DCless-H6-(P)GKY20 purified fusion proteins (lanes 7, 9, 11, respectively), and hydrolyzed proteins (lane 8, 10, 12, respectively). Onc-P: Onconase/Peptide fusion protein; Onc: Onconase carrier; P: (P)GKY20 peptide. Fragments obtained by hydrolysis of ONC-(P)GKY20 fusion protein (B, lane 6) are numbered as in Fig 3.

When Asp-Cys sequences were changed to Asp-Tyr [ONC(YY)-(P)GKY20 fusion protein] or to Glu-Tyr [ONC(EYEY)-(P)GKY20 fusion protein] a drastic reduction of unwanted acid cleavage in 0.1 M acetic acid pH 2.0 at 60°C for 24 h was observed (Fig 5A and 5B, respectively). The SDS-PAGE analysis of the cleavage products of ONC(YY)-(P)GKY20 and ONC(EYEY)-(P)GKY20 was similar but not identical. In particular only ONC(YY)-(P)GKY20 reproducibly showed a smear corresponding to bands 2 and 3 in the analysis of ONC-(P)GKY20 (Fig 5A), thus suggesting that only mutation Asp-Cys to Glu-Tyr abolished the cleavage at the bond Asp67-Cys68. On the contrary both mutants showed two close bands (Fig 5A and 5B, respectively) whose migration is similar to that of band 4, attributed to the cleavage at the bond Asp18-Cys19. As mentioned in the previous section the disappearance of high molecular weight bands suggested the release of unwanted peptides produced during the acid cleavage step from contaminant *E*. *coli* proteins (Fig 5B). Therefore, we decided to increase the purity of the protein sample subjected to acid hydrolysis by the addition of a H6 tag sequence to the chimeric proteins. ONC(EYEY)-H6-(P)GKY20 mutant was produced and purified as described in Materials and Methods. As it is shown in Fig 5, the addition of the H6 tag significantly improved the purity of the chimeric protein from about 80% (Fig 5B) to more than 96–98% as determined by SDS-PAGE densitometry scan (Fig 5C), with a recovery of about 95% of the total fusion protein. Purified ONC(EYEY)-H6-(P)GKY20 was subjected to acid cleavage in 0.1 M acetic acid pH 2.0 at 60°C for 24 h as described above. SDS-PAGE analysis of ONC(EYEY)-H6-(P)GKY20 acid cleavage mixtures (Fig 5C) still evidenced the presence of the two faint bands observed in the case of ONC(EYEY)-(P)GKY20 thus demonstrating that these bands derive from cleavage of the fusion proteins and not from the contaminants. Taken together, our data suggested that, in addition to the bonds Asp18-Cys19 and Asp67-Cys68, further acid labile bonds are present in Onconase. Studies on protein and peptide aging have demonstrated that all Asp-X bonds can undergo spontaneous hydrolysis, even if at a lesser extent than the Asp-Pro bond [38–40]. For example, Joshi and co-workers [39, 40] demonstrated that at acidic pH glucagon undergoes degradation due to hydrolysis at the Asp9-Tyr10 and Asp15-Ser16 bonds and at a lesser extent at the Asp21-Phe22 bond. These findings are in agreement with our conclusion that Asp67-Tyr68 in ONC(YY)-(P)GKY20 fusion protein contributes to the release of ONC derived fragments and suggest that one or more of the additional Asp-X sequences present in the ONC moiety (Asp2-Trp3, Asp16-Val17, Asp20-Asn21 and Asp30-Lys31) could be sensitive to acid hydrolysis. In particular one or both the two close bands observed in the hydrolysis products of ONC(YY)-(P)GKY20 and ONC(EYEY)-(P)GKY20 fusion protein could be tentatively attributed to cleavage at the Asp16-Val17 and/or Asp20-Asn21 and/or Asp-30-Lys31 bonds.

### Improvement of the carrier sequence: second generation mutants

Mutations introduced into the second generation protein ONC-DCless-H6-(P)GKY20 were chosen to remove all the potential unwanted cleavage sites and to reduce the possibility of unwanted formation of intra-chain disulfides without changing the expression level of the fusion protein, its propensity to form inclusion bodies in *E*. *coli* and the propensity of denatured Onconase to form large aggregates at pH higher than 5 as this feature is very useful to remove the carrier after the acid cleavage by means of a simple centrifugation step (as described below). To this aim, as discussed for the first generation mutants, the four remaining aspartic acid residues in the ONC moiety were replaced with glutamic acid residues to preserve the charge and all the cysteine residues were mutated to other residues of similar or higher hydrophobicity chosen on the basis of the secondary structure of native ONC. Accordingly, the single cysteine residue (Cys48) present within alpha helix 3 (S2 File) was replaced with a leucine residue (a “helix-preferring” residue). The two cysteine residues located in beta strands (Cys87 and Cys90) were replaced with isoleucine (a “beta-preferring” residue). Finally, two cysteine residues located in loops (Cys30 and Cys75) were replaced with tyrosine residues. It should be noted that the mutation of cysteine to leucine or isoleucine increases significantly the hydrophobicity and, likely, the propensity of the protein to aggregate.

ONC-DCless-H6-(P)GKY20 (Table 1), was produced in the form of inclusion bodies with a yield similar or higher than that observed for ONC-(P)GKY20 (Fig 2), thus demonstrating that the amino acid substitutions into the carrier sequence did not influence protein expression levels. After purification on Ni-sepharose, ONC-DCless-H6-(P)GKY20 showed about 98% purity as determined by SDS-PAGE densitometry scan (Fig 5D), with a recovery of about 95%.

After cleavage in 0.1 M acetic acid adjusted at pH 2.0, at 60°C for 24 h, SDS-PAGE analysis allowed to estimate 95% efficiency in the release of (P)GKY20. It is worth noting that no unwanted cleavage was observed, thus confirming that aspartic acid residues were responsible for the low efficiency cleavages observed in the case of ONC(EYEY)-H6-(P)GKY20 protein (Fig 5D).

**Fig 6:**
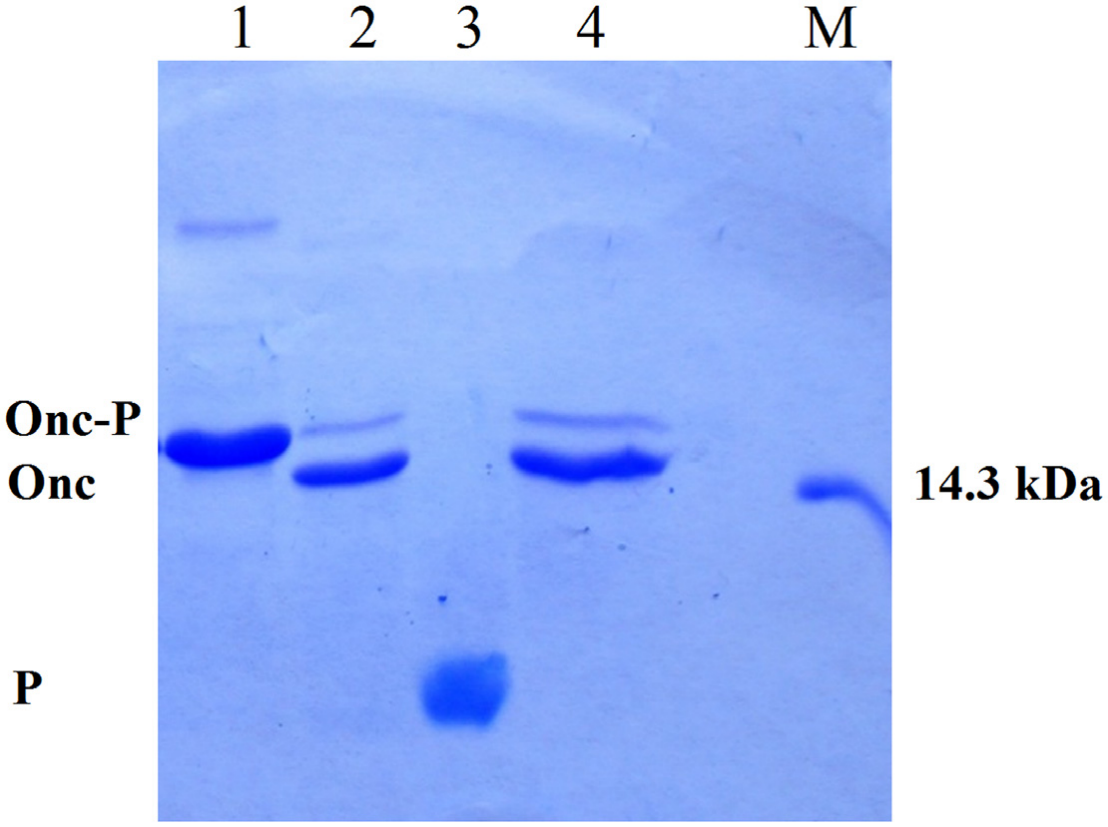
Purification of recombinant (P)GKY20 peptide. DS-PAGE (20%) analysis. Lane M: marker (14.3 kDa). Lane 1: ONC-DCless-H6-(P)GKY20 fusion protein purified by IMAC. Lane 2: cleaved fusion protein (0.1 M acetic acid adjusted at pH 2.0 with HCl, 60°C, 24 h). Lane 3: soluble fraction after neutralization of the cleavage reaction [(P)GKY20 peptide]. Lane 4: insoluble fraction after neutralization of the cleavage reaction (uncleaved fusion protein and Onconase cleaved carrier). Onc-P: Onconase/Peptide fusion protein; Onc: Onconase carrier; P: (P)GKY20 peptide.

**Fig 7:**
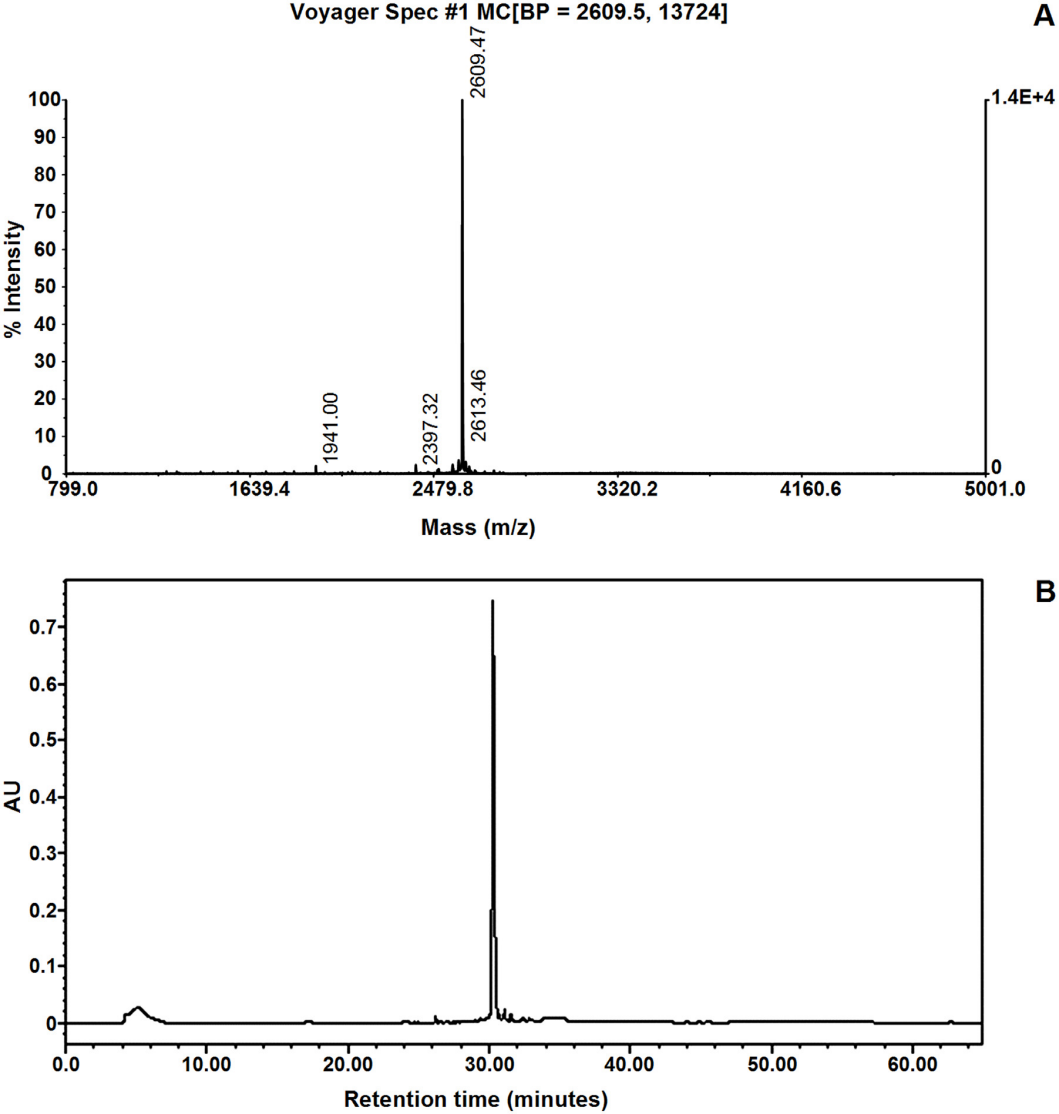
Characterization of purified recombinant (P)GKY20 peptide. (A) Mass spectrum of purified (P)GKY20 peptide. The measured molecular weight (2609.47 Da) is consistent with the theoretical value (2609.1 Da). (B) Reverse-phase HPLC chromatogram recorded at 280 nm wavelength. Purified peptide was applied to a C18 column (Jupiter 5u C18 300Å, 250 x 4.6 mm) and eluted with a linear gradient from 5% to 95% acetonitrile containing 0.05% trifluoroacetic acid, over 60 min at flow rate of 1 mL/min.

Adjusting the pH to 7.2–7.4 with NH_3_ and incubating the mixture at 28°C for 16 h the uncleaved ONC-DCless-H6-(P)GKY20 and ONC-DCless-H6 proteins formed macroscopic insoluble aggregates which were completely removed by centrifugation (Fig 6). Peptide (P)GKY20 was recovered in the soluble fraction, lyophilized and used for the characterization described below without any further purification step. Mass spectrometry analysis (Fig 7A), confirmed the peptide identity and showed that no modified form (e.g. oxidized or formylated peptides) was present in the sample.

Final purity of (P)GKY20 peptide, as determined by reverse phase chromatography (Fig 7B), typically ranged from 95% to 99%.

The optimized procedure allowed the purification of about 10–11 mg of peptide starting from 100 mg of purified fusion protein with 70–75% recovery efficiency (actual milligrams of peptide/expected milligrams of peptide) with respect to the theoretical amount of peptide in the fusion protein.

### Cleavage by cyanogen bromide of ONC-DCless-H6-(PM)GKY20 fusion protein

Starting from ONC-DCless-H6-(P)GKY20 we prepared mutant ONC-DCless-H6-(PM)GKY20. In this fusion protein the additional mutation, a single amino acid insertion, is located in the peptide moiety rather than in the ONC moiety which is identical to that of ONC-DCless-H6-(P)GKY20. By acid hydrolysis it releases peptide (PM)GKY20 carrying two additional residues at the N-terminus. However, as ONC-DCless-H6 does not contain methionine residues, ONC-DCless-H6-(PM)GKY20 fusion protein can also be cleaved using CNBr to release the peptide GKY20 which does not have any additional residue at the N-terminus.

**Fig 8:**
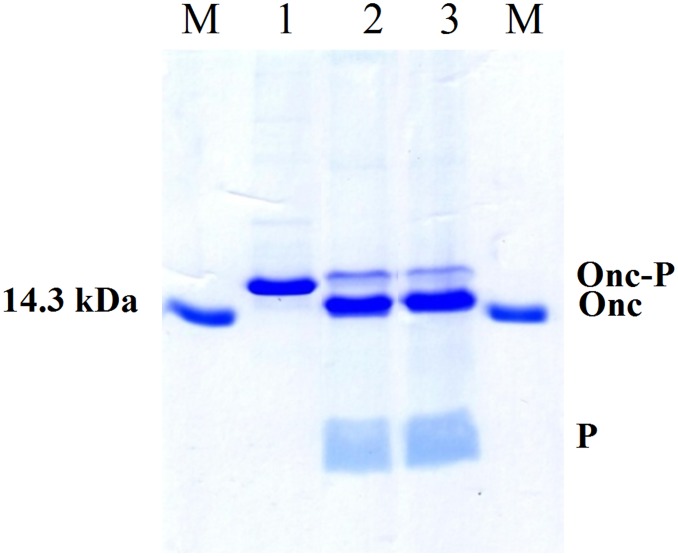
Asp-Pro cleavage efficiency comparison of ONC-DCless-H6-(P)GKY20 and ONC-DCless-H6-(PM)GKY20 fusion proteins. Samples were cleaved in 0.1 M acetic acid adjusted at pH 2.0 with HCl (60°C, 24 h) and analyzed on 20% SDS-PAGE. Lane M: marker (14.3 kDa). Lane 1: ONC-DCless-H6-(PM)GKY20 purified fusion protein. Lanes 2, 3: ONC-DCless-H6-(PM)GKY20 (lane 2) and ONC-DCless-H6-(P)GKY20 (lane 3) hydrolyzed proteins. Onc-P: Onconase/Peptide fusion protein; Onc: Onconase carrier; P: (P)GKY20 peptide.

By acid cleavage ONC-DCless-H6-(PM)GKY20 provided results identical to those obtained in the case of ONC-DCless-H6-(P)GKY20 (Fig 8), thus showing that the additional methionine residue following the Asp-Pro sequence has no influence on the acid cleavage efficiency.

**Fig 9:**
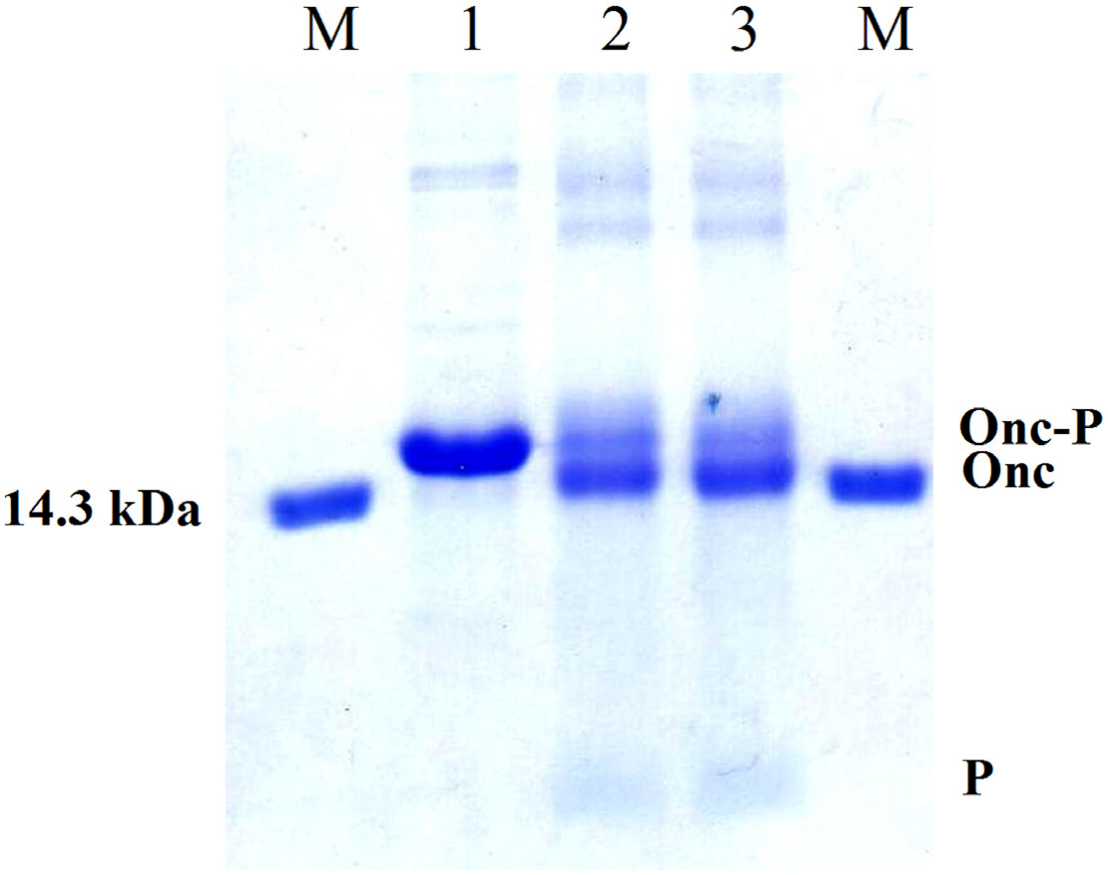
Cyanogen bromide cleavage of ONC-DCless-H6-(PM)GKY20 fusion protein. Samples were analyzed on 20% SDS-PAGE. Lane M: marker (14.3 kDa). Lane 1, purified fusion protein. Lanes 2, 3: cleaved recombinant protein in 0.2 M HCl with 100- (lane 2) and 400-fold (lane 3) molar excess of CNBr over methionine residues. Sample were incubated in the dark at room temperature for 24 h. Onc-P: Onconase/Peptide fusion protein; Onc: Onconase carrier; P: (P)GKY20 peptide.

ONC-DCless-H6-(PM)GKY20 fusion protein was also cleaved by using CNBr in 0.2 M HCl as described in details in the Material and Methods section. As shown in Fig 9 the efficiency of cleavage (about 80%, as determined by SDS-PAGE densitometry scan) was only slightly lower than that obtained with acid hydrolysis, thus demonstrating that ONC-DCless-H6-(PM)GKY20 fusion protein can be effectively cleaved by both chemical methods.

### Antimicrobial activity

**Fig 10:**
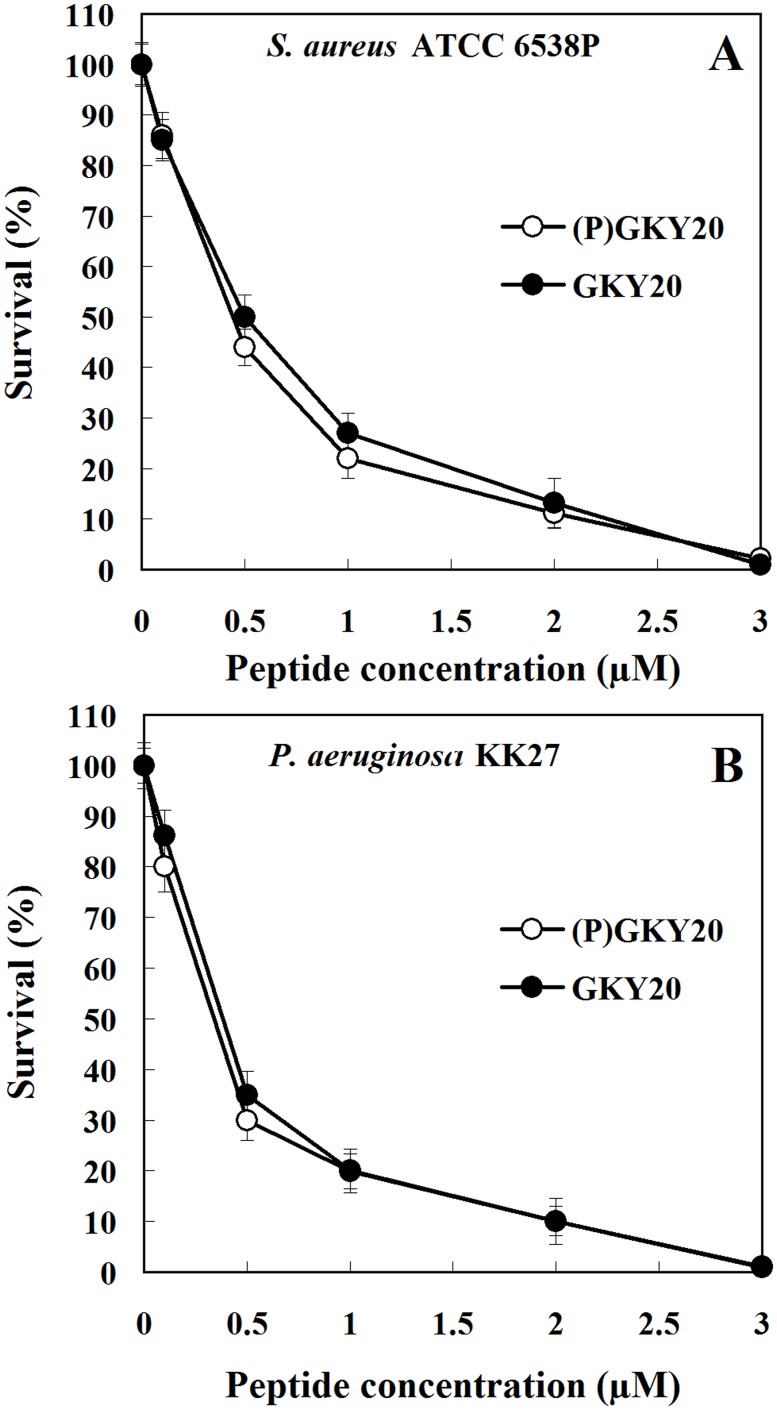
Antibacterial activity of recombinant (P)GKY20 and synthetic GKY20 peptides. Assays were carried out by viable-count method. (A) *Staphylococcus aureus* ATCC 6538P; (B) *Pseudomonas aeruginosa* KK27. Dose-effect curves: 0.1–0.5-1-2-3 μM final concentrations were tested. Error bars are standard deviations. Black circles: synthetic GKY20. White circles: (P)GKY20.

In order to verify if recombinant peptide (P)GKY20 possesses antimicrobial activity comparable to that of a synthetic GKY20, both peptides were tested by plate viable-count assay on a Gram-positive strain, *Staphylococcus aureus* ATCC 6538P, and a Gram-negative strain, *Pseudomonas aeruginosa* KK27 [a clinical strain isolated from a cystic fibrosis patient kindly provided by Dr. Alessandra Bragonzi (San Raffaele Hospital, Milan)]. As shown in Fig 10, the antimicrobial activity of (P)GKY20 was identical to that measured for synthetic GKY20.

**Fig 11:**
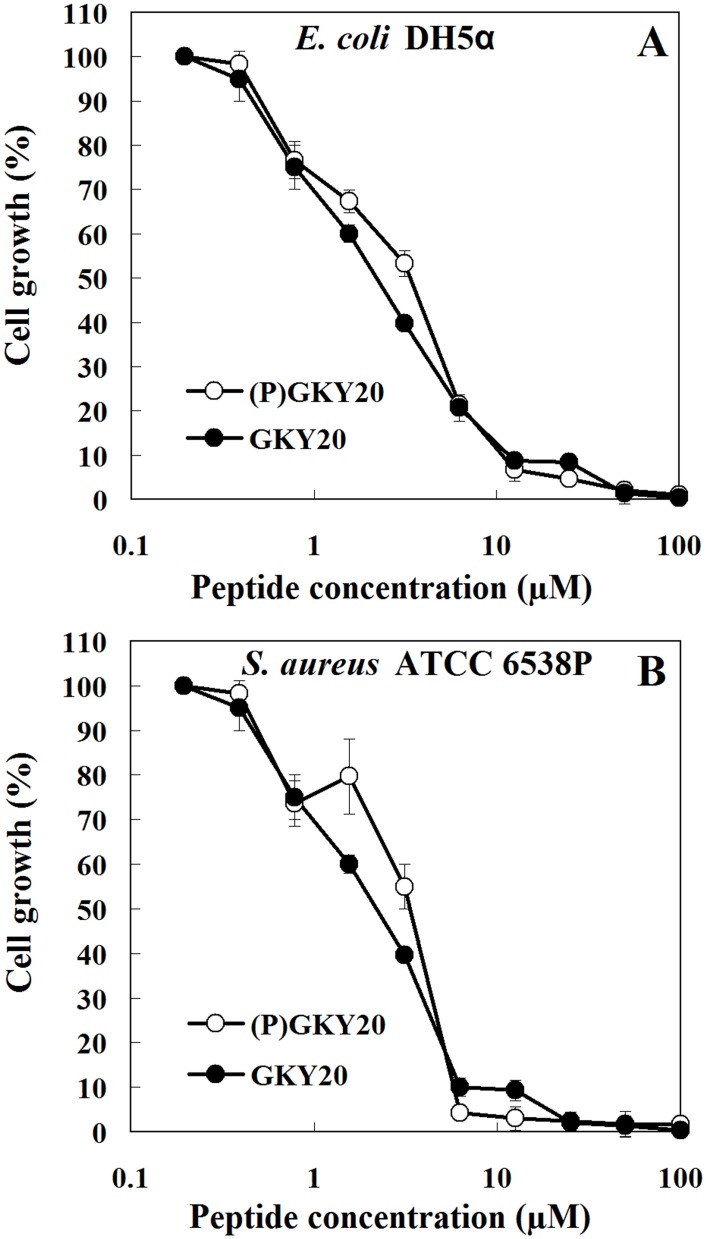
Inhibition growth assay of *E*. *coli* DH5α (A) and *S*. *aureus* ATCC 6538P (B). Assays were carried out by broth dilution method in Nutrient Broth 0.5x. Bacteria from overnight culture were inoculated (~5x10^5^ CFU/mL) in the presence of different concentration of tested peptides (0.2–100 μM). After incubation at 37°C for 16 h, the minimum inhibitory concentration (MIC) was determined as the lowest concentration showing no visible growth. All measurements were carried out three times in independent experiments.

Moreover, to further investigate if N-terminal proline of (P)GKY20 affected the potency of the peptide, we determined, by broth microdilution method, the MIC values (the lowest concentration of antimicrobial agent at which no growth is observed) of the two peptides. As shown in Table 2 and Fig 11, peptides exhibited the same MIC values towards several Gram-positive and Gram-negative strains. These data are very close to those determined against *E*. *coli* and *S*. *aureus* strains for synthetic C-terminal human thrombin peptides [33]. Taken together, these findings suggest that N-terminal proline residue does not change the antimicrobial properties of recombinant (P)GKY20.

**Table 2:** Antibacterial activity of Thrombin derived peptides.

	MIC (μM)^a^					
	(P)GKY20			GKY20		
Bacterial strain	Replicates^b^			Replicates^b^		
***E*. *coli* DH5α**	12.5	12.5	12.5	12.5	12.5	12.5
***E*. *coli* ATCC 25922**	12.5	12.5	25	12.5	12.5	25
***E*. *coli* ATCC 35218**	12.5	12.5	12.5	12.5	12.5	12.5
***P*. *aeruginosa* PAO1**	25	12.5	25	25	12.5	25
***P*. *aeruginosa* PA14**	50	50	25	50	50	25
***S*. *aureus* ATCC 6538P**	6.25	3.12	6.25	6.25	3.12	6.25
***K*. *pneumoniae* ATCC 700603**	12.5	12.5	12.5	12.5	12.5	12.5
***B*. *megaterium* SF185**	12.5	12.5	12.5	12.5	12.5	12.5

^a^Assays were carried out by broth dilution method in Nutrient Broth 0,5 X.

^b^Replicates were from three independent experiments.

## Conclusions

Through a rational design strategy we have progressively improved the suitability of ONC as a carrier for the production of recombinant peptides. The final version of the protein, ONC-DCless-H6, may likely be a very efficient carrier particularly suited for the production of peptides expected to be very toxic for the host *E*. *coli* like antimicrobial peptides. It is expressed at high levels (>200 mg/L) efficiently forming inclusion bodies, and thus sequestering the hosted peptide in an insoluble form. Once solubilized by chaotropic agents the chimeric protein can be easily purified by Ni-chelate chromatography without the need of removing the denaturing agent. At pH lower than 4 denatured ONC is very soluble, therefore chemical cleavage procedures that require an acidic environment (e.g. cleavage of aspartyl-prolyl bonds and CNBr cleavage) can be performed also in diluted acids such as 0.1 M acetic acid or HCl without the need to employ high concentrations of formic acid that is often chosen for its denturing/solubilizing properties [28, 41] but, as already mentioned, causes several unwanted reactions. After the cleavage at acidic pH, the ONC moiety, ONC-DCless-H6, can be easily removed due to its complete insolubility at neutral pH. This feature may allow a simple and effective purification of any peptide that is soluble at pH 7.0. Alternatively, the carrier could be removed under denaturing conditions by Ni-chelate chromatography as after acidic cleavage it retains the H6 tag. Table 3 shows a comparison between our method and those reported in some recent papers describing the production of recombinant antimicrobial peptides.

**Table 3:** Recently published papers reporting the production of recombinant antimicrobial peptides in *Escherichia coli* (*E*.*c*.) or *Bacillus subtilis* (*B*.*s*.).

Reference	Host	Carrier^a^	number of peptides	Carrier features	Purification of fusion proteins^i^	Cleavage method (efficiency)	Peptide Purification^i^	Peptide yield	Peptide purity
This paper	*E*.*c*.	ONC-DCless-H6	2^b^	Inclusion Bodies forming/pH dependent solubility	Inclusion Bodies isolation & I.M.A.C.	Diluted acid at 60°C for 18 h (>95%) or CNBr (~80%)^j^	Selective precipitation	~ 15 to 18 mg/L (~ 13 to 16 mg/g of dry cells)	>95%
[51]	*E*.*c*.	DAMP4var	2^c^	Highly soluble artificial thermostable protein	Two selective precipitation steps	Diluted acid at 60°C for 48 h (high)	Selective precipitation	Not reported	Not reported^m^
[52]	*E*.*c*.	His tagged ketosteroid isomerase	2^d^	Inclusion Bodies forming	IB isolation & I.M.A.C.	thrombin (80%-95%)^k^	I.M.A.C.	30 mg/g of dry cells	>95%
[53]	*E*.*c*.	His tagged SUMO^*a*^	1^e^	Soluble	I.M.A.C.	SUMO protease (complete)	I.M.A.C.	Not reported	>95%
[54]	*B*.*s*.	His tagged SUMO^*a*^	1^f^	Soluble	I.M.A.C.	SUMO protease cleavage	I.M.A.C.	5.5 mg/L (0.16 mg/g of wet cells^l^)	>94%
[55]	*E*.*c*.	Chitin binding domain/Intein fusion^*a*^	1^g^	Soluble/chitin binding/auto-proteolytic	Adsorption to chitin beads (at 4°C)	auto-proteolysis at 25°C for 16 h (~50%)	RP-HPLC	1.7 mg/L	Not reported
[56]	*B*.*s*.	His tagged thioredoxin^*a*^	1^h^	Soluble/Secreted	selective precipitation/I.M.A.C	enterokinase (complete)	I.M.A.C./Cation exchange	2 mg/L	~ 92%

^*a*^His tagged ketosteroid isomerase, SUMO, CBD/Intein and thioredoxin-based methods are commercially available; here we report the most recent applications based on these carriers.

^*b*^Sequences of peptides (P)GKY20 and (PM)GKY20 are in S1 File.

^*c*^PS-Pexiganan-HH (PS-Pex-HH; PS-GIGKFLKKAKKFGKAFVKILKK-HH) and omiganan (GILRWPWWPWRRK-HHHHHH). Omiganan was expressed as fusion protein but not further analysed.

^*d*^p53pAnt (GSRAHSSHLKSKKGQSTSRHKKWKMRRNQFWVKVQRG) and PNC27 (PPLSQETFSDLWKLLKKWKMRRNQFWVKVQRG).

^*e*^Scorpine (GWINEEKIQKKIDERMGNTVLGGMAKAIVHKMAKNEFQCMANMDMLGNCEKHCQTSGEKGYCHGTKCKCGTPLSY).

^*f*^Plectasin (GFGCNGPWDEDDMQCHNHCKSIKGYKGGYCAKGGFVCKCY).

^*g*^P11-5 (GKLFKKILKIL)

^*h*^PR39, (RRRPRPPYLPRPRPPPFFPPRLPPRIPPGFPPRFPPRFP).

^*i*^Dialysis and lyophilization steps are not indicated.

^*j*^Measured only for ONC-DCless-H6-(PM)GKY20.

^*k*^Only peptide p53pAnt could be cleaved by thrombin. 80% cleavage efficiency was obtained by incubating for 48h the fusion protein with thrombin, whereas, 95% efficiency was reached by addition of fresh enzyme and further incubation.

^*l*^Calculated from data in figure 3 of reference 4.

^*m*^Authors state that after the selective precipitation “…most of the DAMP4var was removed by centrifugation (Fig 7A, lane 2), and relatively pure PS-Pex-HH was obtained for MIC testing (Fig 7A, lane 3 and Fig 7B)”.

As for the cleavage of the hosted peptide, ONC-DCless-H6 is very versatile. As it does not contain methionine residues nor Asp-Pro sequences or any other acid sensitive sequence, hosted peptides may be released either by CNBr or by acidic cleavage. Furthermore, as the ONC scaffold does not contain any cysteine residues, peptides may also be released by using 2-nitro-5-thiocyanatobenzoic acid, a reagent that very specifically modify thiols causing the cleavage of X-Cys bonds [20, 28], by replacing the DP or DPM sequences at the end of the flexible linker with a cysteine.

Our strategy, due to its simplicity and limited costs, is also suited for the scale up and could thus be useful for medium/large scale production of peptides needed, for example, in the clinical trial phases.

## Materials and Methods

### Materials

Expression host strain *E*. *coli* BL21(DE3) and plasmid pET22b(+) were purchased from Novagen (San Diego, CA, USA). *E*. *coli* strain TOP10F’ was obtained from Invitrogen (San Diego, CA, USA). QIAprep spin miniprep kit was from Qiagen (Germantown, MD, USA). Wizard SV Gel and PCR Clean-Up DNA Purification System for elution of DNA fragments from agarose gel was purchased from Promega (Madison, WI, USA). Enzymes and other reagents for DNA manipulation were from New England Biolabs (Ipswich, MA, USA). Ni Sepharose^™^ 6 Fast Flow was from GE Healthcare (Uppsala, Sweden). The GKY20 peptide was chemically synthesized by INBIOS s.r.l. (University of Naples, Italy). Difco Nutrient Broth was from Becton-Dickenson (Franklin Lakes, NJ). All other chemicals were from Sigma-Aldrich (Milano, Italy).

### General procedures

Bacterial cultures, plasmid purifications and DNA manipulation were carried out according to Sambrook [42]. DNA sequences and oligonucleotide synthesis were performed by Eurofins MWG Operon service (Ebersberg, Germany). Sodium Dodecyl Sulphate PolyAcrylamide Gel Electrophoresis (SDS-PAGE) was carried out according to Laemmli [43]. *Gallus gallus* lysozyme (14.3 kDa), human pancreatic RNase A (13.8 kDa), recombinant Onconase from *Rana pipiens* (11.8 kDa) and RK-16 peptide from *Salmo salar* Ss-RNasi 1 [44] (16 aa: RYPHCRYRGSPPSTRK; theoretical molecular weight, 1961.25 Da) were used as molecular markers. When appropriate, the relative amount of bands was determined by densitometry performed using the Gel Doc^™^ XR system (Bio-Rad Laboratories, Inc.) equipped with the Quantity One^®^ Software. Protein concentration was determined by the Bradford Protein Assay (Sigma-Aldrich, St. Louis, MO, USA) and standard curves were generated using bovine serum albumin (BSA). Concentrations of purified fusion proteins and peptides were determined by spectrophotometric analysis using the extinction coefficients calculated using the ProtParam tool (accessible to the address http://web.expasy.org/protparam/) [45]. Amino-terminal sequencing was carried out on polypeptides separated by denaturing gel electrophoresis and then electroblotted onto polyvinylidene difluoride (PVDF) membranes [46].

### Construction of the expression vectors

DNA sequences coding for fusion proteins reported in Table 1, were obtained by chemical synthesis (MWG-Biotech AG; Ebersberg, Germany) or by PCR mutagenesis.

Synthetic genes coding for recombinant proteins ONC-(P)GKY20, ONC-DCless-H6-(P)GKY20 and ONC-DCless-H6-(PM)GKY20 (Table 1) were obtained by MWG-Biotech AG (Ebersberg, Germany). All codons were optimized for expression in *E*. *coli* and restriction sites *NdeI* and *SacI* were introduced at 5’- and 3’-end of the synthetic genes, respectively, for their cloning into pET22b(+) vector (Fig 1; S1 File).

All other DNA sequences coding for mutants reported in Table 1 were obtained by Overlap Extension PCR Mutagenesis or by PCR Mutagenesis [47] using the appropriate primers and *pET22b(+)/ONC-(P)GKY20* or *pET22b(+)/ONC(EYEY)-(P)GKY20* plasmids as templates. PCR reactions were carried out using the Phusion High Fidelity DNA Polymerase (Thermo Fisher, New England Biolabs). PCR reactions were started by preheating samples at 94°C for 2 min. Amplifications were performed as follows: 20 cycles at 94°C for 30 s, 56°C for 30 s and 72°C for 30 s, with a final elongation stage at 72°C for 10 min. Amplified products were digested with the appropriate restriction enzymes for their cloning into the pET22b(+) vector. *E*. *coli* strain TOP10F’ was used for all cloning procedures.

### Expression of recombinant proteins

*E*. *coli* strain BL21(DE3) was used to express recombinant proteins. Cells, transformed with pET recombinant plasmids, were grown in 10 mL of Terrific Broth medium (TB) containing 100 μg/ml ampicillin, at 37°C up to OD_600nm_ of 2. These cultures were used to inoculate 1 L of TB/ampicillin medium. Glucose at final concentration of 4 g/L was added to cultures to limit protein expression before induction with IPTG. TB medium was found to be able to induce protein expression over the early growth phase reducing the final optical density of cultures and therefore the recombinant protein yield [48]. Cultures were incubated at 37°C up to OD_600nm_ of 3.5–4. Expression of recombinant proteins was induced by addition of IPTG (isopropyl-β-D-thiogalactopyranoside) at a final concentration of 0.4 mM. Cells were harvested after overnight induction by centrifugation at 8000x *g* for 15 min at 4°C and washed with 50 mM Tris-HCl buffer, pH 7.4. The bacterial pellet was suspended in 50 mM Tris-HCl buffer, pH 7.4, containing 10 mM ethylenediaminetetraacetic acid (EDTA), and sonicated in a cell disruptor (10 x 1 min cycle, on ice). The suspension was then centrifuged at 18,000 x *g* for 60 min at 4°C. Soluble and insoluble fractions were analyzed by SDS-PAGE. The insoluble fractions containing the recombinant protein as inclusion bodies, were washed three times in 0.1 M Tris-HCl buffer, pH 7.4, containing 10 mM EDTA, 2% Triton X-100 and 2 M urea, followed by repeated washes in 0.1 M Tris-HCl buffer, pH 7.4, to eliminate traces of Triton, urea and EDTA. This procedure eliminated several contaminant proteins and cellular debris entrapped in inclusion body pellets.

### Purification of fusion proteins

To purify fusion proteins lacking His_6_-Tag, a procedure was developed to obtain partially purified protein without any chromatographic step. To this purpose, 100 mg of fusion proteins were dissolved in 10 mL of denaturing buffer (6 M guanidine/HCl in 50 mM Tris-HCl, pH 7.4) containing 10 mM β-mercaptoethanol. Mixtures were incubated at 37°C for 3 h under nitrogen atmosphere on a rotary shaker, and then centrifuged at 18,000 x *g* for 60 min at 4°C. Soluble fractions were collected by centrifugation and acidified to pH 3.0 by adding glacial acetic acid before extensive dialysis against 0.1 M acetic acid at 4°C. Soluble fusion proteins were separated from insoluble *E*. *coli* proteins by centrifugation and stored at -80°C under nitrogen atmosphere.

His_6_-tagged recombinant proteins were purified by immobilized metal ion affinity chromatography (IMAC), using the Ni Sepharose^™^ 6 Fast Flow resin. 100 mg of fusion proteins were dissolved in 10 mL of denaturing buffer containing 10 mM β-mercaptoethanol and incubated on a rotary shaker at 37°C for 3 h under nitrogen atmosphere. Soluble fractions were collected by centrifugation and incubated with 5 mL of Ni Sepharose^™^ 6 Fast Flow resin equilibrated in denaturing buffer. The resin was shaken at 4°C for 16 h and then collected by centrifugation. The supernatant, containing the unbound proteins, was discarded. The resin was washed three times with 25 ml of denaturing buffer at 4°C for 30 min and then packed in a glass column. The fusion proteins were eluted with 20 ml of 0.1 M sodium acetate buffer, pH 5.0, containing 6 M guanidine/HCl (elution buffer). The eluate was extensively dialyzed against 0.1 M acetic acid at 4°C. Samples were stored at -80°C under nitrogen atmosphere. Purified fusion protein concentrations were determined by spectrophotometric analysis using the extinction coefficients calculated using the ProtParam tool [ε_280_ = 24,410 M^_1^ cm^_1^; ε_280_ (0.1%) = 1.53].

### Cleavage optimization of Asp-Pro peptide bond

Fusion proteins were cleaved by thermal incubation in acid solutions under nitrogen atmosphere. To optimize chemical cleavage, formic acid and acetic acid, were tested. Purified fusion proteins in 0.1 M acetic acid were lyophilized and suspended in i) 50%, 60% and 70% formic acid or ii) 0.1 M acetic acid at pH 2.9 or at pH 2.0 (by addition of diluted HCl). The mixtures were incubated for 3-6-9-15-24 h, at 37°C, 50°C and 60°C. Cleavage efficiency was analyzed by SDS-PAGE using 20% gel. Formaldehyde was used to covalently link proteins and polypeptides in polyacrylamide gels [49]. To this purpose, 4% formaldehyde was added to the Coomassie Blue staining solution (0.2% Coomassie Brilliant Blue R-250, 25% isopropyl alcohol and 10% glacial acetic acid). Percentage of cleaved protein was determined by densitometric analysis of SDS-PAGE gels as described in “General procedures”.

Identification of polypeptides obtained by chemical cleavage was carried out by N-terminal sequencing. Proteins and polypeptides were separated by denaturing gel electrophoresis, electroblotted onto polyvinylidene difluoride (PVDF) membranes, stained by Ponceau S dye and sequenced by the Edman method [46].

### Chemical cleavage and purification of recombinant (P)GKY20 peptide

Cleavage of the peptide from fusion proteins was performed in 0.1 M acetic acid at pH 2.0, incubating samples at 60°C for 24 h in a water bath under nitrogen atmosphere. The mixtures were then neutralized to pH 7–7.2 by adding NH_3_, purged with N_2_ and incubated at 28°C for 16 h in a water bath. The soluble peptide was isolated from the insoluble carrier through repeated cycles of centrifugation at 18,000 x g for 60 min at 4°C. Purified peptide was lyophilized and stored at -80°C. Peptide concentrations were determined by spectrophotometric analysis using the extinction coefficients calculated using the ProtParam tool [ε_280_ = 8,480 M^-1^ cm^-1^; recombinant (P)GKY20 peptide, ε_280_ (0.1%) = 3.250; GKY20 peptide, ε_280_ (0.1%) = 3.376].

The purity of (P)GKY20 peptide was verified by SDS-PAGE and reverse-phase HPLC using a Waters (Milford, MA, USA) HPLC system (1525 binary pump and 2996 photodiode array detector). The column was a C18 (250 x 4,6 mm, 5μm particle size) Jupiter 5u C18 300 Å (Phenomenex). The solvents were 0.05% trifluoracetic acid (TFA) in water (solvent A) and 0.05% TFA in acetonitrile (solvent B). The column was equilibrated with 5% acetonitrile and the peptide was eluted by a linear gradient ranging from 5% to 95% solvent B over 60 min, at a flow rate of 1 ml/min. The elution was monitored at 280 nm. Purity was determined by measuring the relative area of peaks using the Empower software (Waters, Milford, MA, USA).

The identity of the peptide was determined by mass spectrometry. Positive Reflectron MALDI spectra were recorded on a Voyager DE STR instrument (AB Sciex, Framingham, MA). The MALDI matrix was prepared by dissolving α-cyano-hydroxycinnamic powder in 70% acetonitrile and 30% citric acid 50 mM. Typically 1 μL of matrix was applied to the metallic sample plate and 1 μL of analyte was added. The mixture thus obtained was then dried at room temperature. Acceleration and reflector voltages were set up as follows: target voltage at 20 kV, first grid at 65% of target voltage, delayed extraction at 400 ns to obtain the best signal-to-noise ratios and the best possible isotopic resolution. Mass calibration was performed using external peptide standards purchased from AB Sciex. Each spectrum represents the sum of 3,000 laser pulses from randomly chosen spots per sample position. Raw data were analyzed using the computer software provided by the manufacturers and are reported as monoisotopic masses.

### Cyanogen bromide cleavage reaction

CNBr cleavage was performed on purified recombinant ONC-DCless-H6-(PM)GKY20 protein. Sample was lyophilized and then resuspended in 0.2 M HCl at final concentration of 1.5 mg/mL. Cleavage was started by the addition of 100- and 400-fold molar excess of CNBr (40 mg/mL in 0.2 M HCl stock solution) over methionine residues, and the mixtures were incubated in the dark at room temperature for 24 h. Reaction mixtures were dried under vacuum and washed three times in 0.1 M acetic acid to allow CNBr evaporation. Samples were resuspended in 0.1 M acetic acid and the efficiency of cleavage was estimated by densitometric analysis of SDS-PAGE.

### Antimicrobial assay

Antibacterial activity assays were carried out by i) agar dilution plate viable-count method [44] and ii) broth microdilution method for antimicrobial peptides [50].

Agar dilution plate viable-count method was performed as previously described [44] with minor modifications. A single colony of *Pseudomonas aeruginosa* (KK27) or *Staphylococcus aureus* (ATCC 6538P) was suspended in 5 mL of Luria-Bertani (LB) medium (Becton, Dickinson) and overnight incubated at 37°C. When the cultures reached an OD_600nm_ of 1 unit, they were diluted 1:100 in 20 mM sodium phosphate buffer (NaP), pH 7.0. Samples were prepared by adding 40 μL of bacterial cells to peptides at 0.1, 0.5, 1.0, 2.0, and 3.0 μM final concentrations, in 1 mL of 20 mM NaP buffer, pH 7.0. Cells incubated with antibiotic (colistin 0.01 mg/mL and ampicillin 0.05 mg/mL) were used as positive control, whereas cells incubated without any antibiotics or peptide were prepared as negative control. Bovine serum albumin (BSA) was tested at the same concentration of the peptides. Samples were incubated at 37°C for 4 hours and dilutions (1:100 and 1:1000) of all the samples were placed on LB/agar medium and incubated overnight at 37°C. The following day survived cells were estimated by colonies counting on each plate and compared with controls. All compounds were tested in triplicate experiments. Standard deviations were always less than 5%.

Minimal Inhibitory Concentration (MIC) was determined by broth microdilution method following the protocol previously described for antimicrobial peptides [50] with minor modifications. Assays were carried out in Difco Nutrient Broth composed of 0.5% beef extract (w/v), 0.05% pepton and 0.25% NaCl, using sterile 96-well polypropylene microtiter plates (cat. 3879,Costar Corp., Cambridge, MA). Twofold serial dilutions of peptides were carried out in the test wells to obtain concentrations ranging from 100 μM to 0.2 μM. Bacteria were inoculated from an overnight culture at a final concentration of ~ 5x10^5^ CFU/mL per well and incubated overnight at 37°C. MIC value was taken as the lowest concentration at which growth was inhibited. Three independent experiments were performed for each MIC value. Controls included the peptide antibiotic polymyxin B and vancomycin (Sigma, St. Louis, MO). MIC values were measured on: *Escherichia coli* DH5α, *P*. *aeruginosa* PAO1, *P*. *aeruginosa* PA14, *B*. *megaterium* SF185, *S*. *aureus* ATCC 6538P from our laboratory strains collection and on *Escherichia coli* ATCC 25922, *Escherichia coli* ATCC 35218, *K*. *pneumoniae* ATCC 700603 (kindly provided by Eliana De Gregorio, University of Naples Federico II, Italy).

## Supporting Information

S1 File Nucleotide and amino acid sequences. (A) ONC-(P)GKY20 and (B) ONC-DCless-H6-(P)GKY20 fusion proteins. Onconase carrier (black); peptide (blue); linker region (red underlined); His_6_-Tag (green). Amino acid substitutions in ONC-DCless-H6-(P)GKY20 protein sequence (B) were highlighted: glutamate residues (red), tyrosine residues (purple), leucine residue (light blue), isoleucine residues (orange). The main restriction enzyme sites were also reported: *NdeI* (turquoise); *EcoRI* (yellow); *KpnI* (grey); *BamHI* (green); *SacI* (pink). (C) Sequence alignment of human prothrombin gene (GenBank: M17262) segment coding for GKY20 (black) and the manually improved sequence (blue). Mutated codons were underlined and nucleotide substitutions were highlighted in yellow.
(DOCX)

S2 File Onconase secondary structure. Alpha helices (h) and beta strands (e) from Onconase crystallographic structure [PDB:1ONC] are shown below the sequence. (A) Amino acid sequence of ONC mutant (Onconase M23L, C104 delete); (B) amino acid sequence of ONC-DCless mutant (Onconase M23L, D2E, D16E, D18E, C19Y, D20E, C30Y, D32E, C48L, D67E, C68Y, C75Y, C87I, C90I, C104 delete). The amino acid substitutions are pointed out: red, D2E, D16E, D18E, D20E, D32E, D67E mutations; grey, M23L mutation; turquoise, C19Y, C30Y, C68Y, C75Y mutations; light green, C48L mutation; dark green, C87I, C90I mutations; yellow, cysteine residues.
(DOCX)

## References

[1] Wiesner J, Vilcinskas A. Antimicrobial peptides: the ancient arm of the human immune system. Virulence. 2010;1(5):440–64. Epub 2010/12/24. 10.4161/viru.1.5.12983 . 21178486

[2] Fjell CD, Hiss JA, Hancock RE, Schneider G. Designing antimicrobial peptides: form follows function. Nat Rev Drug Discov. 2012;11(1):37–51. Epub 2011/12/17. 10.1038/nrd3591 . 22173434

[3] Hilchie AL, Wuerth K, Hancock RE. Immune modulation by multifaceted cationic host defense (antimicrobial) peptides. Nat Chem Biol. 2013;9(12):761–8. Epub 2013/11/16. 10.1038/nchembio.1393 . 24231617

[4] Gaspar D, Veiga AS, Castanho MA. From antimicrobial to anticancer peptides. A review. Front Microbiol. 2013;4:294 Epub 2013/10/09. 10.3389/fmicb.2013.00294 24101917 PMC3787199

[5] Reissmann S. Cell penetration: scope and limitations by the application of cell-penetrating peptides. J Pept Sci. 2014;20(10):760–84. Epub 2014/08/13. 10.1002/psc.2672 . 25112216

[6] Li X, Chen W, Zhan Q, Dai L, Sowards L, Pender M, et al Direct measurements of interactions between polypeptides and carbon nanotubes. J Phys Chem B. 2006;110(25):12621–5. Epub 2006/06/28. 10.1021/jp061518d . 16800593

[7] Katoch J, Kim SN, Kuang Z, Farmer BL, Naik RR, Tatulian SA, et al Structure of a peptide adsorbed on graphene and graphite. Nano Lett. 2012;12(5):2342–6. Epub 2012/04/05. 10.1021/nl300286k . 22471315

[8] Ingham AB, Moore RJ. Recombinant production of antimicrobial peptides in heterologous microbial systems. Biotechnol Appl Biochem. 2007;47(Pt 1):1–9. Epub 2007/04/17. 10.1042/BA20060207 . 17432953

[9] Zorko M, Jerala R. Production of recombinant antimicrobial peptides in bacteria. Methods Mol Biol. 2010;618:61–76. Epub 2010/01/23. 10.1007/978-1-60761-594-1_5 . 20094858

[10] Li Y. Recombinant production of antimicrobial peptides in *Escherichia coli*: a review. Protein Expr Purif. 2011;80(2):260–7. Epub 2011/08/17. 10.1016/j.pep.2011.08.001 . 21843642

[11] Li Y. Carrier proteins for fusion expression of antimicrobial peptides in *Escherichia coli*. Biotechnol Appl Biochem. 2009;54(1):1–9. Epub 2009/07/07. 10.1042/BA20090087 . 19575694 PMC7188355

[12] Zhou QF, Luo XG, Ye L, Xi T. High-level production of a novel antimicrobial peptide perinerin in *Escherichia coli* by fusion expression. Curr Microbiol. 2007;54(5):366–70. Epub 2007/05/09. 10.1007/s00284-006-0466-y . 17486407

[13] Moon JY, Henzler-Wildman KA, Ramamoorthy A. Expression and purification of a recombinant LL-37 from *Escherichia coli*. Biochim Biophys Acta. 2006;1758(9):1351–8. Epub 2006/03/18. 10.1016/j.bbamem.2006.02.003 . 16542635

[14] Beaulieu L, Tolkatchev D, Jette JF, Groleau D, Subirade M. Production of active pediocin PA-1 in *Escherichia coli* using a thioredoxin gene fusion expression approach: cloning, expression, purification, and characterization. Can J Microbiol. 2007;53(11):1246–58. Epub 2007/11/21. 10.1139/w07-089 . 18026219

[15] Wu G, Deng X, Li X, Wang X, Wang S, Xu H. Application of immobilized thrombin for production of S-thanatin expressed in *Escherichia coli*. Appl Microbiol Biotechnol. 2011;92(1):85–93. Epub 2011/06/10. 10.1007/s00253-011-3379-z . 21655979

[16] Feng XJ, Wang JH, Shan AS, Teng D, Yang YL, Yao Y, et al Fusion expression of bovine lactoferricin in *Escherichia coli*. Protein Expr Purif. 2006;47(1):110–7. Epub 2005/10/12. 10.1016/j.pep.2005.08.016 . 16216526

[17] Li Y, Li X, Li H, Lockridge O, Wang G. A novel method for purifying recombinant human host defense cathelicidin LL-37 by utilizing its inherent property of aggregation. Protein Expr Purif. 2007;54(1):157–65. Epub 2007/03/27. 10.1016/j.pep.2007.02.003 . 17382559

[18] Gibbs GM, Davidson BE, Hillier AJ. Novel expression system for large-scale production and purification of recombinant class IIa bacteriocins and its application to piscicolin 126. Appl Environ Microbiol. 2004;70(6):3292–7. Epub 2004/06/09. 10.1128/AEM.70.6.3292-3297.2004 15184123 PMC427731

[19] Vidovic V, Prongidi-Fix L, Bechinger B, Werten S. Production and isotope labeling of antimicrobial peptides in *Escherichia coli* by means of a novel fusion partner that enables high-yield insoluble expression and fast purification. J Pept Sci. 2009;15(4):278–84. Epub 2009/02/04. 10.1002/psc.1112 . 19189273

[20] Crimmins DL, Mische SM, Denslow ND. Chemical cleavage of proteins in solution. Curr Protoc Protein Sci. 2005;Chapter 11:Unit 11 4. Epub 2008/04/23. 10.1002/0471140864.ps1104s40 . 18429104

[21] Bornstein P, Balian G. Cleavage at Asn-Gly bonds with hydroxylamine. Methods Enzymol. 1977;47:132–45. Epub 1977/01/01. . 927171 10.1016/0076-6879(77)47016-2

[22] Li Y, Li X, Wang G. Cloning, expression, isotope labeling, and purification of human antimicrobial peptide LL-37 in *Escherichia coli* for NMR studies. Protein Expr Purif. 2006;47(2):498–505. Epub 2005/12/06. 10.1016/j.pep.2005.10.022 . 16325420

[23] Tian ZG, Dong TT, Yang YL, Teng D, Wang JH. Expression of antimicrobial peptide LH multimers in *Escherichia coli* C43(DE3). Appl Microbiol Biotechnol. 2009;83(1):143–9. Epub 2009/02/12. 10.1007/s00253-009-1893-z . 19205689

[24] Zorko M, Japelj B, Hafner-Bratkovic I, Jerala R. Expression, purification and structural studies of a short antimicrobial peptide. Biochim Biophys Acta. 2009;1788(2):314–23. Epub 2008/11/26. 10.1016/j.bbamem.2008.10.015 . 19026609

[25] Ryan RO, Forte TM, Oda MN. Optimized bacterial expression of human apolipoprotein A-I. Protein Expr Purif. 2003;27(1):98–103. Epub 2003/01/03. 10.1016/S1046-5928(02)00568-5 . 12509990

[26] Montigny C, Penin F, Lethias C, Falson P. Overcoming the toxicity of membrane peptide expression in bacteria by upstream insertion of Asp-Pro sequence. Biochim Biophys Acta. 2004;1660(1–2):53–65. Epub 2004/02/06. 10.1016/j.bbamem.2003.10.013 . 14757220

[27] Landon. Cleavage at aspartyl-prolyl bonds. Methods Enzymol. 1977;47:145–9. Epub 1977/01/01. . 22018 10.1016/0076-6879(77)47017-4

[28] Hwang PM, Pan JS, Sykes BD. Targeted expression, purification, and cleavage of fusion proteins from inclusion bodies in *Escherichia coli*. FEBS Lett. 2014;588(2):247–52. Epub 2013/10/01. 10.1016/j.febslet.2013.09.028 . 24076468

[29] Notomista E, Cafaro V, Fusiello R, Bracale A, D'Alessio G, Di Donato A. Effective expression and purification of recombinant onconase, an antitumor protein. FEBS Lett. 1999;463(3):211–5. Epub 1999/12/22. 10.1016/S0014-5793(99)01623-3 . 10606723

[30] Notomista E, Catanzano F, Graziano G, Dal Piaz F, Barone G, D'Alessio G, et al Onconase: an unusually stable protein. Biochemistry. 2000;39(30):8711–8. Epub 2000/07/29. 10.1021/bi000415x . 10913282

[31] Papareddy P, Rydengard V, Pasupuleti M, Walse B, Morgelin M, Chalupka A, et al Proteolysis of human thrombin generates novel host defense peptides. PLoS Pathog. 2010;6(4):e1000857 Epub 2010/04/28. 10.1371/journal.ppat.1000857 20421939 PMC2858699

[32] Merza M, Rahman M, Zhang S, Hwaiz R, Regner S, Schmidtchen A, et al Human thrombin-derived host defense peptides inhibit neutrophil recruitment and tissue injury in severe acute pancreatitis. Am J Physiol Gastrointest Liver Physiol. 2014;307(9):G914–21. Epub 2014/09/13. 10.1152/ajpgi.00237.2014 . 25214403

[33] Kasetty G, Papareddy P, Kalle M, Rydengard V, Morgelin M, Albiger B, et al Structure-activity studies and therapeutic potential of host defense peptides of human thrombin. Antimicrob Agents Chemother. 2011;55(6):2880–90. Epub 2011/03/16. 10.1128/AAC.01515-10 21402837 PMC3101415

[34] Notomista E, Catanzano F, Graziano G, Di Gaetano S, Barone G, Di Donato A. Contribution of chain termini to the conformational stability and biological activity of onconase. Biochemistry. 2001;40(31):9097–103. Epub 2001/08/02. 10.1021/bi010741s . 11478876

[35] Singh A, Upadhyay V, Upadhyay AK, Singh SM, Panda AK. Protein recovery from inclusion bodies of *Escherichia coli* using mild solubilization process. Microb Cell Fact. 2015;14:41 Epub 2015/04/19. 10.1186/s12934-015-0222-8 25889252 PMC4379949

[36] Wang L. Towards revealing the structure of bacterial inclusion bodies. Prion. 2009;3(3):139–45. Epub 2009/10/07. 10.4161/pri.3.3.9922 19806034 PMC2802778

[37] Mant CT, Kovacs JM, Kim HM, Pollock DD, Hodges RS. Intrinsic amino acid side-chain hydrophilicity/hydrophobicity coefficients determined by reversed-phase high-performance liquid chromatography of model peptides: comparison with other hydrophilicity/hydrophobicity scales. Biopolymers. 2009;92(6):573–95. Epub 2009/10/02. 10.1002/bip.21316 19795449 PMC2792893

[38] Li N, Fort F, Kessler K, Wang W. Factors affecting cleavage at aspartic residues in model decapeptides. J Pharm Biomed Anal. 2009;50(1):73–8. Epub 2009/04/28. 10.1016/j.jpba.2009.03.020 . 19395214

[39] Joshi AB, Kirsch LE. The estimation of glutaminyl deamidation and aspartyl cleavage rates in glucagon. Int J Pharm. 2004;273(1–2):213–9. Epub 2004/03/11. 10.1016/j.ijpharm.2004.01.006 . 15010145

[40] Joshi AB, Sawai M, Kearney WR, Kirsch LE. Studies on the mechanism of aspartic acid cleavage and glutamine deamidation in the acidic degradation of glucagon. J Pharm Sci. 2005;94(9):1912–27. Epub 2005/07/30. 10.1002/jps.20405 . 16052557

[41] Houen G, Svaerke C, Barkholt V. The solubilities of denatured proteins in different organic solvents. Acta Chem Scand. 1999;53(12):1122–6. Epub 2000/01/12. . 10629937 10.3891/acta.chem.scand.53-1122

[42] Green MR, Hughes H, Sambrook J, MacCallum P, editors. Molecular Cloning: A Laboratory Manual. 4th ed: Cold Spring Harbor laboratory Pess; 2012.

[43] Laemmli UK. Cleavage of structural proteins during the assembly of the head of bacteriophage T4. Nature. 1970;227(5259):680–5. Epub 1970/08/15. . 5432063 10.1038/227680a0

[44] Pizzo E, Varcamonti M, Di Maro A, Zanfardino A, Giancola C, D'Alessio G. Ribonucleases with angiogenic and bactericidal activities from the Atlantic salmon. FEBS J. 2008;275(6):1283–95. Epub 2008/02/19. 10.1111/j.1742-4658.2008.06289.x . 18279393

[45] Gasteiger E, Hoogland C, Gattiker A, Duvaud S, Wilkins MR, Appel RD, et al Protein Identification and Analysis Tools on the ExPASy Server. In: Walker JM, editor. The Proteomics Protocols Handbook Humana Press 2005 p. 571–607.

[46] Matsudaira P. Sequence from picomole quantities of proteins electroblotted onto polyvinylidene difluoride membranes. J Biol Chem. 1987;262(21):10035–8. Epub 1987/07/25. . 3611052

[47] Lee J, Shin MK, Ryu DK, Kim S, Ryu WS. Insertion and deletion mutagenesis by overlap extension PCR. Methods Mol Biol. 2010;634:137–46. Epub 2010/08/03. 10.1007/978-1-60761-652-8_10 . 20676981

[48] Pan SH, Malcolm BA. Reduced background expression and improved plasmid stability with pET vectors in BL21 (DE3). Biotechniques. 2000;29(6):1234–8. Epub 2000/12/29. . 11126126 10.2144/00296st03

[49] Steck G, Leuthard P, Burk RR. Detection of basic proteins and low molecular weight peptides in polyacrylamide gels by formaldehyde fixation. Anal Biochem. 1980;107(1):21–4. Epub 1980/09/01. 10.1016/0003-2697(80)90486-8 . 6159805

[50] Wiegand I, Hilpert K, Hancock RE. Agar and broth dilution methods to determine the minimal inhibitory concentration (MIC) of antimicrobial substances. Nat Protoc. 2008;3(2):163–75. Epub 2008/02/16. 10.1038/nprot.2007.521 . 18274517

[51] Zhao CX, Dwyer MD, Yu AL, Wu Y, Fang S, Middelberg AP. A simple and low-cost platform technology for producing pexiganan antimicrobial peptide in E. coli. Biotechnol Bioeng. 2015;112(5):957–64. Epub 2014/11/27. 10.1002/bit.25505 . 25425208

[52] Rodriguez V, Asenjo JA, Andrews BA. Design and implementation of a high yield production system for recombinant expression of peptides. Microb Cell Fact. 2014;13:65 Epub 2014/06/03. 10.1186/1475-2859-13-65 24885242 PMC4022411

[53] Zhang C, He X, Gu Y, Zhou H, Cao J, Gao Q. Recombinant scorpine produced using SUMO fusion partner in *Escherichia coli* has the activities against clinically isolated bacteria and inhibits the *Plasmodium falciparum* parasitemia in vitro. PLoS One. 2014;9(7):e103456 Epub 2014/07/30. 10.1371/journal.pone.0103456 25068263 PMC4113386

[54] Zhang L, Li X, Wei D, Wang J, Shan A, Li Z. Expression of plectasin in *Bacillus subtilis* using SUMO technology by a maltose-inducible vector. J Ind Microbiol Biotechnol. 2015;42(10):1369–76. Epub 2015/08/25. 10.1007/s10295-015-1673-y . 26299602

[55] Basu A, Mishra B, Dey S, Leong SSJ. Intein based bioprocess for production of a synthetic antimicrobial peptide: an alternative route to solid phase peptide synthesis. RSC Adv. 2014;4:31564–72. 10.1039/C4RA04056B

[56] Wang J, Tan T, Li X, Zhang L, Feng X, Shan A, et al Recombinant secretory expression, purification and antimicrobial activity of PR39 in *Bacillus subtilis* using a maltose-inducible vector. Process Biochem. 2015 10.1016/j.procbio.2015.08.005

