# Supplementary material for: Rational Design of a Carrier Protein for the Production of Recombinant Toxic Peptides in Escherichia coli
Source: PLoS One. 2016 Jan 25;11(1):e0146552. doi: 10.1371/journal.pone.0146552 (PMC4726619; doi:10.1371/journal.pone.0146552)
Supplement: S1 File — (A) ONC-(P)GKY20 and (B) ONC-DCless-H6-(P)GKY20 fusion proteins. Onconase carrier (black); peptide (blue); linker region (red underlined); His6-Tag (green). Amino acid substitutions in ONC-DCless-H6-(P)GKY20 protein sequence (B) were highlighted: glutamate residues (red), tyrosine residues (purple), leucine residue (light blue), isoleucine residues (orange). The main restriction enzyme sites were also reported: NdeI (turquoise); EcoRI (yellow); KpnI (grey); BamHI (green); SacI (pink). (C) Sequence alignment of human prothrombin gene (GenBank: M17262) segment coding for GKY20 (black) and the manually improved sequence (blue). Mutated codons were underlined and nucleotide substitutions were highlighted in yellow. (DOCX) [file pone.0146552.s001.docx]

1. **ONC-(P)GKY20**

# NdeI

**CATATG**CAAGACTGGCTGACTTTCCAGAAAAAACATATCACTAACACTCGTGACGTTGAC

M Q D W L T F Q K K H I T N T R D V D

TGCGACAACATCCTGTCTACTAACCTGTTCCATTGCAAAGACAAAAACACTTTCATCTAC

C D N I L S T N L F H C K D K N T F I Y

TCTCGTCCGGAACCGGTTAAAGCTATCTGCAAAGGTATCATCGCTTCTAAAAACGTTCTG

S R P E P V K A I C K G I I A S K N V L

**EcoRI**

ACTACTTCT**GAATTC**TACCTGTCTGACTGCAACGTTACTTCTCGTCCGTGCAAATACAAA

T T S E F Y L S D C N V T S R P C K Y K

CTGAAAAAATCTACTAACAAATTCTGCGTTACTTGCGAAAACCAGGCTCCGGTTCATTTC

L K K S T N K F C V T C E N Q A P V H F

**KpnI BamHI** **(P)GKY20**

GTTGGTGTTGGTTCT**GGTACC**GG**GGATCC**Gggcaaatatggcttctacacccatgtgttc

V G V G S G T G D P G K Y G F Y T H V F

**(P)GKY20 SacI**

cgcctgaaaaaatggattcagaaagtcattTAA**GAGCTC**

R L K K W I Q K V I -

1. **ONC-DC*less*-H6-(P)GKY20**

# NdeI

**CATATG**CAA**GAA**TGGCTGACTTTCCAGAAAAAACATATCACTAACACTCGT**GAA**GTT**GAA**

M Q **E** W L T F Q K K H I T N T R **E** V **E**

**TATGAA**AACATCCTGTCTACTAACCTGTTCCAT**TAT**AAA**GAA**AAAAACACTTTCATCTAC

**Y** **E** N I L S T N L F H **Y** K **E** K N T F I Y

TCTCGTCCGGAACCGGTTAAAGCTATC**CTG**AAAGGTATCATCGCTTCTAAAAACGTTCTG

S R P E P V K A I **L** K G I I A S K N V L

**EcoRI**

ACTACTTCT**GAATTC**TACCTGTCT**GAATAT**AACGTTACTTCTCGTCCG**TAT**AAATACAAA

T T S E F Y L S **E Y** N V T S R P **Y** K Y K

CTGAAAAAATCTACTAACAAATTC**ATT**GTTACT**ATT**GAAAACCAGGCTCCGGTTCATTTC

L K K S T N K F **I** V T **I** E N Q A P V H F

**KpnI BamHI** **(P)GKY20**

GTTGGTGTTGGTTCTCATCATCATCATCATCAT**GGTACC**GG**GGATCC**Gggcaaatatggc

V G V G S **H H H H H H** G T G D P G K Y G

**(P)GKY20 SacI**

ttctacacccatgtgttccgcctgaaaaaatggattcagaaagtcattTAA**GAGCTC**

F Y T H V F R L K K W I Q K V I -

1. **GKY20 codon optimization**

gggaaatatggcttctacacacatgtgttccgcctgaagaagtggatacagaaggtcatt

ggcaaatatggcttctacacccatgtgttccgcctgaaaaaatggattcagaaagtcatt
