# Supplementary material for: Rational Design of a Carrier Protein for the Production of Recombinant Toxic Peptides in Escherichia coli
Source: PLoS One. 2016 Jan 25;11(1):e0146552. doi: 10.1371/journal.pone.0146552 (PMC4726619; doi:10.1371/journal.pone.0146552)
Supplement: S2 File — Alpha helices (h) and beta strands (e) from Onconase crystallographic structure [PDB:1ONC] are shown below the sequence. (A) Amino acid sequence of ONC mutant (Onconase M23L, C104 delete); (B) amino acid sequence of ONC-DCless mutant (Onconase M23L, D2E, D16E, D18E, C19Y, D20E, C30Y, D32E, C48L, D67E, C68Y, C75Y, C87I, C90I, C104 delete). The amino acid substitutions are pointed out: red, D2E, D16E, D18E, D20E, D32E, D67E mutations; grey, M23L mutation; turquoise, C19Y, C30Y, C68Y, C75Y mutations; light green, C48L mutation; dark green, C87I, C90I mutations; yellow, cysteine residues. (DOCX) [file pone.0146552.s002.docx]

**A**

1 5 10 15 20 25 30 35 40 45 50 55 60 65 70 75 80 85 90 95 100

| | | | | | | | | | | | | | | | | | | | |

MQDWLTFQKKHITNTRDV**DC**DNILSTNLFHCKDKNTFIYSRPEPVKAICKGIIASKNVLTTSEFYLS**DC**NVTSRPCKYKLKKSTNKFCVTCENQAPVHFVGVGS-

hhhhhhhh hhhhh eeeeee hhhhhhhh eeee eeeeeeee eeeeeeee eeeeee eeeeeeee

**helix 1 helix 2 helix 3**

**B**

1 5 10 15 20 25 30 35 40 45 50 55 60 65 70 75 80 85 90 95 100

| | | | | | | | | | | | | | | | | | | | |

MQ**E**WLTFQKKHITNTR**E**V**EYE**NILSTNLFH**Y**K**E**KNTFIYSRPEPVKAI**L**KGIIASKNVLTTSEFYLS**EY**NVTSRP**Y**KYKLKKSTNKF**I**VT**I**ENQAPVHFVGVGS-

hhhhhhhh hhhhh eeeeee hhhhhhhh eeee eeeeeeee eeeeeeee eeeeee eeeeeeee

**helix 1 helix 2 helix 3**
